# ezBIDS: Guided standardization of neuroimaging data interoperable with major data archives and platforms

**DOI:** 10.1038/s41597-024-02959-0

**Published:** 2024-02-08

**Authors:** Daniel Levitas, Soichi Hayashi, Sophia Vinci-Booher, Anibal Heinsfeld, Dheeraj Bhatia, Nicholas Lee, Anthony Galassi, Guiomar Niso, Franco Pestilli

**Affiliations:** 1 Department of Psychology, Department of Neuroscience, Center for Perceptual Systems, Center for Learning and Memory, Center for Aging Population Sciences, University of Texas, Austin, TX 78712 USA; 2 Department of Psychology and Human Development, Peabody College, Vanderbilt University, Nashville, TN 37203 USA; 3 Center for Multimodal Neuroimaging, National Institute of Mental Health, Bethesda, MD USA; 4 Instituto Cajal, CSIC, Madrid, Spain

**Keywords:** Software, Standards, Neuroscience, Psychology

## Abstract

Data standardization promotes a common framework through which researchers can utilize others’ data and is one of the leading methods neuroimaging researchers use to share and replicate findings. As of today, standardizing datasets requires technical expertise such as coding and knowledge of file formats. We present ezBIDS, a tool for converting neuroimaging data and associated metadata to the Brain Imaging Data Structure (BIDS) standard. ezBIDS contains four major features: (1) No installation or programming requirements. (2) Handling of both imaging and task events data and metadata. (3) Semi-automated inference and guidance for adherence to BIDS. (4) Multiple data management options: download BIDS data to local system, or transfer to OpenNeuro.org or to brainlife.io. In sum, ezBIDS requires neither coding proficiency nor knowledge of BIDS, and is the first BIDS tool to offer guided standardization, support for task events conversion, and interoperability with OpenNeuro.org and brainlife.io.

## Introduction

Data standards provide a framework for effective data sharing^1^. Furthermore, data standards can help address replication issues in neuroimaging^2^, by providing a common framework to harmonize data structures^3^. A lack of data standards has downstream effects that can limit data sharing, curation, interoperability, and ultimately scientific reproducibility^4^. Without standards, attempts at data sharing create confusion due to an inability to readily discern the identity, location, and parameters of others’ data.

The Brain Imaging Data Structure (BIDS) was introduced to provide neuroimagers a pathway to robust reproducibility and replication practices via a widely accepted framework for standardizing the description and organization of brain imaging data^5^. The BIDS standard has grown tremendously over the past several years and currently offers standardization specification for multiple neuroimaging modalities, including magnetic resonance imaging^5^, magnetoencephalography^6^, electroencephalogram^7^, intracranial electroencephalogram^8^, positron emission tomography^9^, arterial spin labeling^10^ and microscopy^11^ with many others under active development. The widespread adoption of BIDS can be attributed to the timely response to addressing data-sharing needs for scientific rigor and transparency^5,12^ and to the availability of community data repositories and platforms, such as OpenNeuro.org^13^ and brainlife.io^14^.

Notwithstanding the widespread adoption of BIDS, data standardization remains a challenge for many researchers, as it requires substantial knowledge of the BIDS specification and coding skills. Over the past several years, a multitude of software tools have been developed to assist researchers in the BIDS conversion process. These tools expect varying levels of manual intervention from the user, such as having users provide pieces of code or configuration files, including complex pattern matching statements, for improved BIDS mapping. A handful of tools promote limited manual intervention in the form of “point-and-click” graphical user interfaces (GUI), for example, plugins such as *Osirix, Horos*, the web-based *BIDS Toolbox*^15^, the *fw-heudiconv* package in Flywheel^16^, *pyBIDSconv*, *Biscuit* for MEG data, and *BIDScoin*^17^.

Despite the important features most BIDS-conversion tools provide, there remain both technical and knowledge hurdles that make converting neuroimaging data to BIDS a nontrivial task. Indeed, most BIDS conversion tools available require knowledge of the Unix/Linux command line (terminal) and moderate programming competency for both package installation and use. These requirements present technical barriers for researchers keen on adopting BIDS but who are more interested in specific domain questions and less inclined on learning new unfamiliar workflows. Furthermore, the limited built-in checks available in most BIDS-conversion tools to guide researchers to compliant BIDS datasets add uncertainty and limit adoption of the standard, with great loss for scientific rigor and transparency. To contribute to the adoption of the BIDS standard, it is necessary to develop conversion tools that support data conversions, while lowering the technical barriers of entry to BIDS by reducing the requirements for technical skills, such as coding and knowledge of the details of the standard specification.

In this article, we present ezBIDS, a BIDS-conversion tool that requires neither installation, programming skills, nor knowledge of the Unix terminal and BIDS specification. ezBIDS uses a Software-as-a-Service (SaaS)^18^ model where researchers can upload imaging data to a secure cloud system that hosts pre-installed software. The web-interface of ezBIDS guides users to organize entire sets of neuroimaging data series into BIDS-compliant datasets. Unlike other conversion tools that require users to input code or configuration files denoting the expected mapping (i.e., heuristics) of raw imaging data to BIDS, ezBIDS provides heuristics to the users. ezBIDS provides a guess for the mapping of the necessary information (e.g., *data type*, *suffix*, entity labels) for each image, and then displays the guess in a web-interface that allows users modifications and editing, if necessary. ezBIDS allows either to download data or push data to OpenNeuro.org or brainlife.io, contributing to lowering the barriers of entry to data standardization and sharing, while advancing scientific rigor and reproducibility.

## Results

**Fig. 1:**
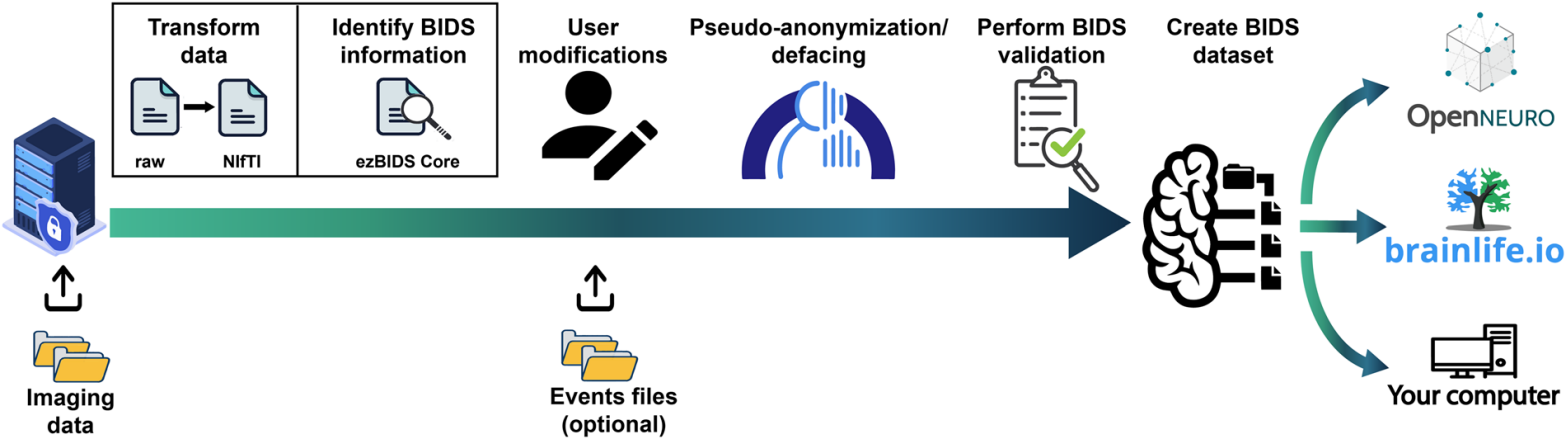
ezBIDS workflow schematic. The ezBIDS schematic overview of the steps necessary to map raw imaging data to BIDS. Users begin by uploading data to a secure ezBIDS server. Once uploaded, several automated backend processes transform the data and identify the relevant information required in BIDS specification. This information is then presented to the user for modifications, if needed. Following this, users may then choose to pseudo-anonymize anatomical data to remove identifying facial features (i.e., deface). ezBIDS then performs a final BIDS validation to ensure compliance with the specification, after which a finalized BIDS dataset is created. Finally, users may download their BIDS dataset to their local computer, or upload it to an open-science repository such as OpenNeuro.org, or to a data analysis platform like brainlife.io. We note that users residing outside of the United States of America (USA) should check local regulations before uploading non pseudonymized data to ezBIDS.

ezBIDS facilitates BIDS conversion through the partnership between humans (user modifications via a web interface) and machines (heuristics to guess the mapping between neuroimaging data and the BIDS specification; Fig. 1).

To ensure a standardized framework, the BIDS specification enforces specific rules dictating the organization and metadata necessary for a proper description of data. Firstly, BIDS requires that data be stored in specific formats. For example, MRI data must be stored in the Neuroimaging Information Technology Initiative file format (NIfTI^19^) and the corresponding metadata must be stored using the JavaScript Object Notation (JSON) format^20^. NIfTI-formatted imaging data is compatible with a broad range of imaging software tools and smaller in size compared to other data formats (e.g., Digital Imaging Communication in Medicine (DICOM)) and JSON-formatted metadata provides relevant information that enables human and machine readability. Once formatted, files must be organized and named in a specific manner to enable the identification of the data. Files are placed in subject (and session, if applicable) folders, followed by *data type* (anatomical, functional, field map, diffusion-weighted imaging, etc.) sub-folders. Inside the *data type* sub-folders, files follow a naming convention that includes an entity labels list, a *suffix*, and a file extension. An entity is an attribute that describes the file, consisting of a hyphenated key-value pair, separated from other entities in the list by an underscore character. A *suffix* (commonly referred to as “modality”), denotes the sub-category of the *data type* (for example, *T1w*, *T2w*, and *FLAIR* are sub-categories of anatomical data). Lastly, the file extension denotes the data format, with *.nii.gz* for NIfTI and *.json* for JSON data. For example, a file named sub-01/ses-01/anat/sub-01_ses-01_T1w.nii.gz indicates that the data is an anatomical (*anat*) T1w sequence collected in the first session (ses-01) of the first subject (sub-01). Furthermore, the corresponding JSON sidecar metadata file (e.g., sub-01/ses-01/anat/sub-01_ses-01_T1w.json) contains data parameters such as the SliceThickness, FlipAngle, ShimSetting, among many others, which provide detailed information regarding its acquisition. In sum, BIDS provides a standardized framework for more effective data sharing and in support of scientific understanding and reproducibility.

The ezBIDS approach for mapping imaging data to BIDS is based on a Propose-and-Revise approach. The first step of this approach entails taking the input imaging data and automatically compiling BIDS-relevant information, which can then be mapped into a candidate BIDS structure (*Propose phase*). The information is gathered via an unsupervised process that uses a set of heuristic functions (see Table S1) implementing a traditional rule-based approach to produce an educated guess for the candidate BIDS-dataset structure given the set of input imaging files. The second phase of the approach is to allow revisions of the *Propose phase* by the user (*Revision phase*), which is presented to the user on the web interface. Drop-down menus and guided selections allow users to make revisions in accordance with the BIDS specification.

The ezBIDS workflow consists of several important steps that assist users in transforming raw imaging (e.g., DICOM) files to a BIDS-compliant dataset. This article reports the functionality of the ezBIDS system from both a user and a developer perspective. In brief, raw imaging data can be uploaded to a secure server hosted by brainlife.io, mapped to the BIDS structure, and then pushed to OpenNeuro.org, brainlife.io, or downloaded back to the user’s computer (Fig. 1).

### Accessing ezBIDS

ezBIDS can be accessed at the URL https://brainlife.io/ezbids (Figure S1). DICOM files or the outputs of dcm2niix^21^ can be uploaded to ezBIDS for guided curation and standardization to BIDS by following the web interface at the ezBIDS URL. ezBIDS web services run on most browsers, but have been primarily tested and developed on Google Chrome, Apple Safari and Firefox. A simple tutorial containing test data is provided for novice users at https://brainlife.io/docs/tutorial/ezBIDS. Technical documentation is provided at https://brainlife.io/docs/using_ezBIDS. Below we describe the technical architecture of ezBIDS and the approach to data standardization and curation.

### Upload raw data files: DICOM or dcm2niix output

ezBIDS has been developed and tested using two types of raw imaging data files: DICOM data as well as NIfTI and JSON data produced by dcm2niix. Data can be uploaded without any specific organizational structure, reducing user efforts for preparing data for conversion. Users may upload compressed imaging data using formats such as.tar.xz,.tar,.tgz,.gz, .7z,.bz2,.zip, and.rar (Figure S2).

Pseudo-anonymized DICOM data upload is supported, however, uploading DICOM data with sufficient unique information to allow mapping subjects and session IDs is preferred. Unique DICOM metadata allows reducing the burden on the users downstream during the conversion process. More specifically, if the metadata fields AcquisitionDateTime, PatientID and PatientName are preserved or at least containing unique information, they can be used by ezBIDS for mapping purposes. These fields typically provide unique subject (and session) information in imaging studies, which ezBIDS uses to organize the data with minimal user input. Note that these fields do not necessarily contain protected health information and it is up to the user to determine whether or not to anonymize the data prior to uploading to ezBIDS. ezBIDS can still process anonymized data lacking these participant-identifying fields but will likely exhibit less efficiency in determining the subject and session mappings, unless this information is explicitly contained (e.g., sub-01) within the file path. Explicit subject and session organization is not required for non-anonymized data, but providing the data organized in subject (and session) specific folders can help to regain some of the efficiency lost after anonymization. Uploaded data are private and non-accessible to other users and protected through the ezBIDS web-based security system (see **Web-security features of ezBIDS** section below). ezBIDS anonymizes the data once the BIDS conversion is complete, such that the data transferred to other cloud systems or downloaded to local computer environments are anonymized.

In lieu of DICOM data, users may instead choose to upload NIfTI and JSON data generated by dcm2niix. This option is convenient in cases where users have lost access to the original DICOM files. However, uploading DICOM data is generally recommended over dcm2niix data, as it contains more metadata facilitating BIDS information extraction, whereas for dcm2niix data there might be a mismatch between the current dcm2niix version used by ezBIDS and the potentially older dcm2niix version used by the user, which might not contain the most up-to-date metadata fields/information. In either case, ezBIDS accepts the data, performs a best-guess mapping to BIDS, and then guides the user through a series of web-based steps to assure that the mapping is accurate. Once uploaded, ezBIDS implements data uncompression if necessary, DICOM-to-NIfTI transformation using dcm2niix (if DICOM data are uploaded), and sends the output to the ezBIDS Core for BIDS (and additional) information identification.

### The ezBIDS Core

**Fig. 2:**
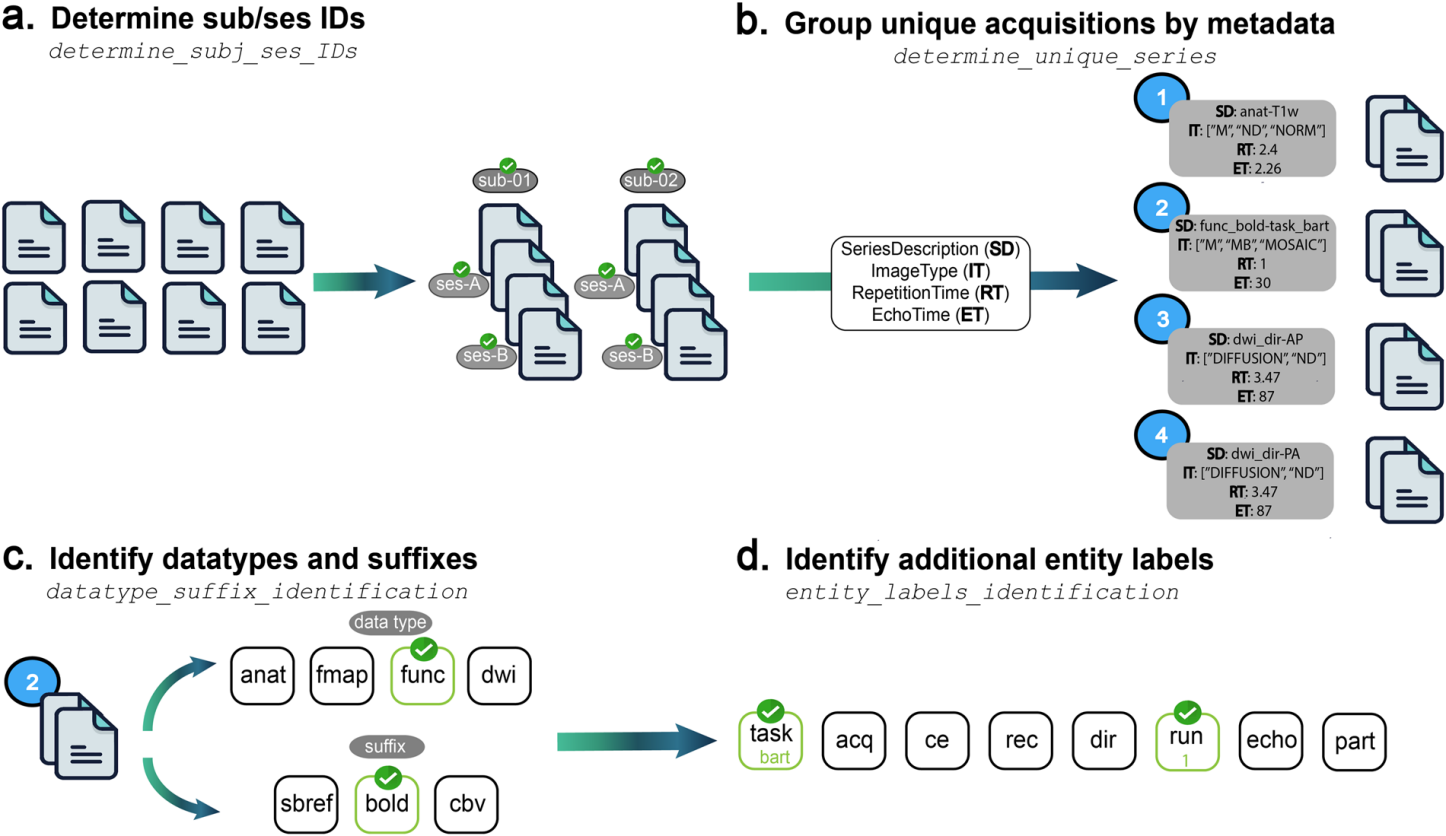
The components of ezBIDS Core. **(a)** Function for determining subject (and session) BIDS entity labels. **(b)** Function for organizing data into unique group series, based on having the same values for the following four metadata fields: SeriesDescription, ImageType, RepetitionTime, EchoTime. **(c)** Function for determining the *data type* and *suffix* BIDS information, which provide the precise identity of the image. **(d)** Function for determining additional BIDS information which provides a better understanding of the image’s purpose.

The ezBIDS Core is a collection of python functions implementing operations to infer the mapping between raw and converted BIDS data files by parsing, interpreting, and organizing the data uploaded (Fig. 2).

**Fig. 3:**
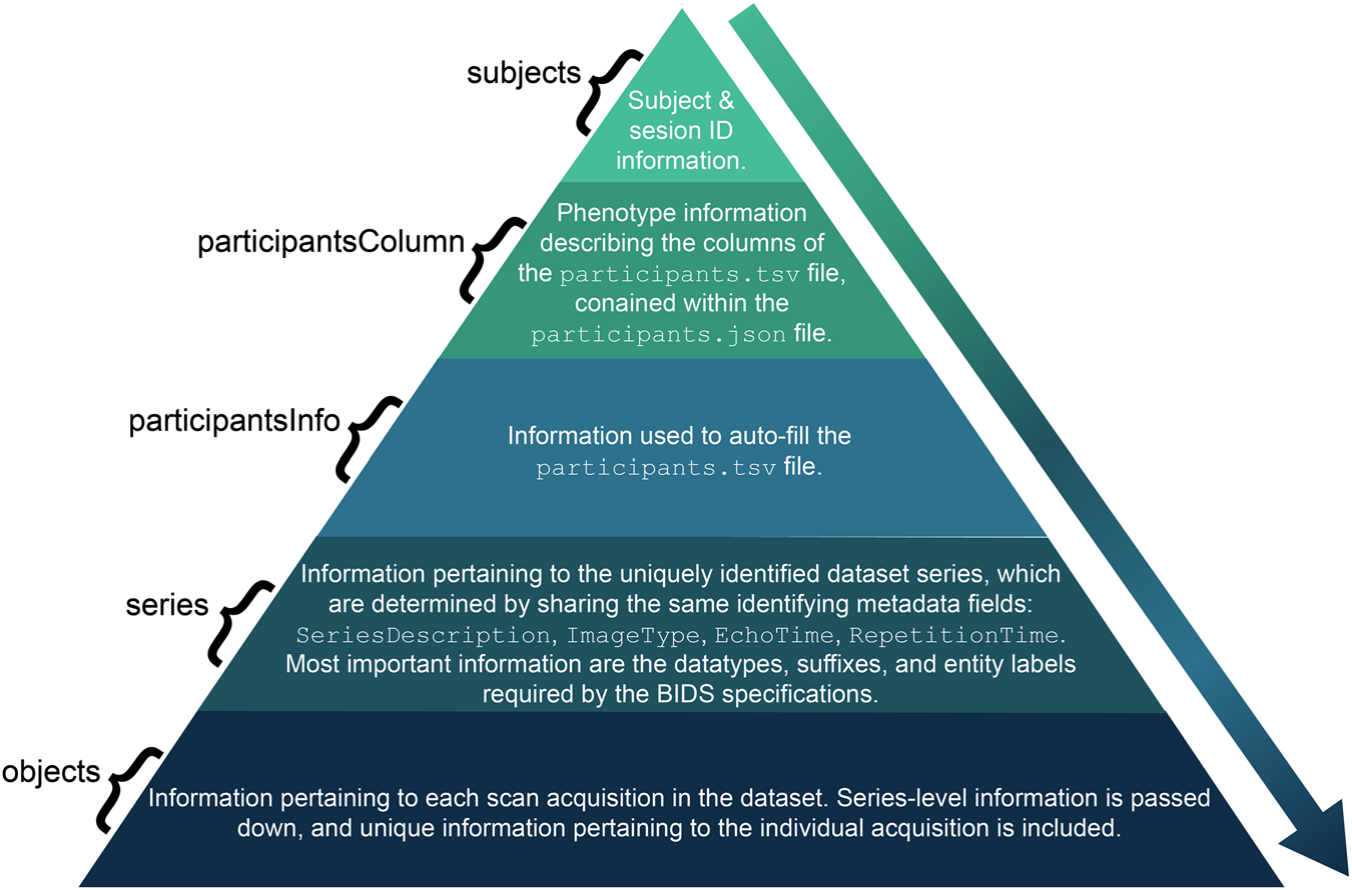
Structure and contents of the ezBIDS_core.json file. Schematic demonstrating the hierarchical structure of the JSON file created by the ezBIDS Core. Each descending section comprises a larger proportion of the ezBIDS_core.json file outputted by ezBIDS. BIDS entity information (sub-, ses-, task-, etc) is passed down to levels of the individual scan sequences (objects). Objects’-level information constitutes the largest proportion of the JSON file, with subject information constituting the least. This JSON file represents an integrated representation of data and metadata mapping DICOM files to BIDS structures.

ezBIDS Core gathers BIDS-relevant and additional information from the imaging data to present to the user via the web interface. ezBIDS Core is unique because it automatically guesses any available information contained within the imaging data provided, with the ultimate product being a JSON file called ezBIDS_core.json, containing metadata and inferred BIDS information (e.g., entity labels; Fig. 3).

The ezBIDS_core.json is used by the ezBIDS web interface to display the BIDS information to the user, enabling edits, approval, and validation. This JSON file is output by the ezBIDS Core without requiring configuration files or predefined file naming conventions. The ezBIDS_core.json can in principle also be used by other informatics systems as it contains BIDS information pertaining to each imaging file mapping, DICOM header information, data screenshots for visualization, and quality assurance information. Below we describe the steps that ezBIDS Core takes to generate the ezBIDS_core.json file.

- *Data transformation*. If DICOM data are uploaded, dcm2niix is executed, transforming the data to NIfTI format. Alternatively, NIfTI and JSON files generated by dcm2niix may be uploaded if the user does not have access to the DICOM files. While rare, any errors generated by the dcm2niix software are logged and presented to the user following this process, informing them which DICOM files(s) triggered an error and advising them to submit an issue on the dcm2niix GitHub page (https://github.com/rordenlab/dcm2niix/issues). Depending on the nature of the error, the offending DICOM(s) may not be transformed into NIfTI files for BIDS conversion. It is recommended that such errors be resolved by reaching out to the dcm2niix team; however, this is not required in order to proceed.
- *Generating BIDS information*. The challenge presented to all BIDS converters is to assign BIDS information to images, such as the data type, *suffix*, and entities (see Table 1 for definitions of these terms). The ezBIDS Core is used to make a best-guess determination of this information, which is done through a series of functions (Table 2). There are four key functions that identify the appropriate BIDS information for each image (Fig. 2).

**Table 1:** Definitions for BIDS-related terms.

Term	Definition
Data type	A functional group of different types of data. Data files are stored in a folder named for the specific *data type* (e.g., *anat*, *func*, *fmap*, etc).
Suffix	The category of brain data recorded by a file. Often referred to as the “modality”, it is a sub-categorization of the *data type* (e.g., *epi* is a *suffix* of the *fmap data type*). The suffix may overlap, but should not be confused with the *data type*.
Entity	Label(s) that helps differentiate the data, based on image parameters (e.g., *acq*, *dir*, etc). Only specific entity labels are allowed for certain *data type* and *suffix* data, and the ordering of entity labels is necessary for BIDS compliance.
Series ID	Unique value given to images based on specific metadata (*SeriesDescription, ImageType*, *RepetitionTime*, and *EchoTime*; see Fig. 2b).
Task events timing files	Files describing timing and other properties of events recorded during a run. For non-rest task functional BOLD images, BIDS highly recommends that such files be formatted as *events.tsv* files, containing standard information such as stimulus *onset*, *duration*, etc.
Validation errors (i.e., bids-validator errors)	Situations in which an entry is not compliant with BIDS specification. Examples include improper entity label(s), misordering of entity labels, use of special characters, etc. Errors must be addressed to ensure BIDS-compliant data.
Validation warnings (additional ezBIDS- specific guidance messages and information)	Situations in which some aspect of the data might be improper and worth addressing. Examples may include alerting users to exclude a sequence (e.g., *events.tsv*) from BIDS conversion because a corresponding sequence (functional BOLD) has been excluded. Warnings are meant to ensure improved quality of the dataset for curation purposes; however, they do not need to be addressed in order to create a BIDS-compliant dataset.

The terms *data type*, *suffix*, *entity*, and *task events timing files* come directly from the BIDS specification.

**Table 2:** Comparison of ezBIDS to other common BIDS converters based on a collection of metrics.

	heudiconv	dcm2bids	bidsify	bidskit	Data2BIDS	BIDScoin	ezBIDS
Command-line interface (CLI)	✓	✓	✓	✓	✓	✓	**✗**
Web interface	✗	✗	✗	✗	✗	✗	**✓**
Active development	✓	✓	✓	✓	✗	✓	**✓**
No coding required	✗	✗	✗	✗	✗	✓	**✓**
Task events timing files conversion	✗	✗	✗	✗	✗	✗	**✓**
QA checks	✗	✗	✗	✗	✗	✗	**✓**
BIDS validation	✗	✓	✗	✗	✗	✓	**✓**
Interoperability with open repositories	✗	✗	✗	✗	✗	✗	**✓**
Collaboration (shareable URLs) with colleagues	✗	✗	✗	✗	✗	✗	**✓**

1. Identify subject and session BIDS entity labels (ezBIDS *determine_subj_ses_IDs*; Fig. 2a). Each image file name is expected to contain the subject entity, as well as the session entity when the individual participant data is acquired over separate scan visits, thus ezBIDS examines each image for this information. The first approach is to find images with unique PatientID, PatientName, and PatientBirthDate metadata values, which are given a unique subject ID starting with “01” and increasing chronologically. However, some researchers will seek to provide anonymized data, where metadata fields pertaining to participant identification are removed, including PatientID, PatientName, and PatientBirthDate. In these scenarios, ezBIDS assigns the subject ID as the folder that the image is stored in, to ensure that data across subjects is not assumed to belong to a single subject. Lastly, ezBIDS uses a regular expression (regex) search pattern to assess whether the subject (and session) ID(s) have been explicitly specified by the researcher. For example, if < sub- > (for subject) and < ses- > (for session) string patterns are found in the file path name, PatientID, or PatientName, then the string following this pattern is assumed to be the subject (or session) ID. Detection of one or more sessions for a particular subject entity entails assessing the AcquisitionDate and AcquisitionTime metadata fields. In accordance with BIDS, a session label is not generated in instances where a subject label contains a single set of AcquisitionDate and AcquisitionTime values unless the < ses-> pattern is detected.
2. Identify and group similar images (ezBIDS *determine_unique_series*; Fig. 2b). Images are grouped together when they contain identical values for the following four metadata fields: SeriesDescription, ImageType, RepetitionTime, and EchoTime. ezBIDS Core assumes that images with these identical parameters are similar enough to require identical BIDS information, such as *data type*, *suffix*, or entity label(s). This grouping procedure enables a more streamlined process and is similar to how the recently published BIDS curation package (CuBIDS) accounts for the heterogeneity of parameters within datasets^22^. Each group of images is assigned a unique series ID, linking it to other images within the group. If ezBIDS or a user adds an entity label (e.g., acq-mprage; Table 1) to a single image, this modification is applied to all other images in the group. Later, users have the ability to make modifications to individual images, enabling more precise control.
  There are two instances in which images can be grouped together while not containing the exact four metadata field values. The first case pertains to slight differences in the SeriesDescription values due to retroactive reconstruction of acquired images. Under circumstances in which normal image reconstruction fails, oftentimes due to insufficient scanner storage space, retroactive reconstruction is required to obtain the data in image space. The string < *_RR* > is typically appended to the end of the SeriesDescription field to denote this procedure, yet the image is functionally the same as other images containing the same SeriesDescription field. The second case pertains to minute divergences in RepetitionTime and EchoTime values, where a slight tolerance is given in instances where these values differ within a+/− 0.5 ms (0.0005 sec) range, to account for slight precision differences in how compilers convert floating-point numbers from binary to ASCII. For example, an image with an EchoTime of 0.03 and another image with an EchoTime of 0.030001 are given the same unique series ID, assuming exact matches on the other metadata values. There are no instances in which grouping by the ImageType does not contain an exact metadata field.
3. Identify *data type* and *suffix* BIDS information for images with unique series ID (ezBIDS *datatype_suffix_identification*; Fig. 2c). A critical component for BIDS conversion is properly determining the *data type* and the *suffix* (also known as the “modality”) of images, which pertains to the identity of the images (Table 1).
  ezBIDS references the BIDS-specification schema, a collection of Yet Another Markup Language (YAML) files containing information regarding the data types accepted by BIDS and the *suffix* labels allowed for each specific *data type*. There exist three heuristics for determining this information. The first entails searching the image’s SeriesDescription metadata field for the existence of an explicit *data type* and/or *suffix* identifier (e.g., *anat_T1w*). The second heuristic entails searching the image’s SeriesDescription metadata field for the existence of common keyphrases used in MRI protocols (see Table S2). For example, “tfl3d” is a commonly used phrase to denote a *T1w* anatomical image, therefore the presence of this phrase provides sufficient information to discern both the *data type* (*anat*) and the *suffix* (*T1w*). The third heuristic entails using additional metadata fields, primarily *ImageType* and *EchoTime*, to discern *data type* and/or *suffix* identities. For example, the phrase “DIFFUSION” in ImageType indicates the presence of diffusion-weighted data (DWI), thus providing evidence regarding the *data type* (*dwi*) and *suffix* (*dwi*) of the image. Additionally, an anatomical image with an EchoTime exceeding 100 ms is assumed to be a *T2w*, thereby identifying the *suffix* (*T2w*). For a description of the metadata fields (ImageType, EchoTime, etc.) and their usage in ezBIDS, see Table S3.
  ezBIDS begins with the first heuristic for determining the *data type* and *suffix* information, proceeding to the subsequent heuristics if this information cannot be discerned with the current heuristic. Should all three heuristic approaches fail to identify the *data type* and *suffix* for an image, its *data type* is set as exclude and left to the user to determine whether the data should be converted, helping to limit ezBIDS misidentifications that users may fail to notice. If the data are to be converted, the user will be asked to specify this information.
4. Identify additional BIDS entity labels (via *entity_labels_identification*; Fig. 2d). A BIDS entity label contains information regarding the data itself and/or its place within the hierarchical BIDS dataset structure. This feature enables users to quickly discern what kind of data is contained within the dataset and its location. Depending on an image’s *data type* and *suffix*, additional entity label information may be required to differentiate images from one another. For example, BIDS requires the phase encoding direction (“dir”) entity label for spin echo field maps. ezBIDS Core performs two passes to determine this information that is achieved through regex search patterns (“_<entityLabel>-”) of SeriesDescription (see Table S2 for examples). Searching other metadata fields can also produce information on the necessary entity labels. For example, dcm2niix produces an EchoNumber metadata field for multi-echo images; the ezBIDS Core looks for this field and if found, adds the integer to the echo entity label.

The overarching goal of the ezBIDS Core automated process is to gather necessary BIDS information and provide a first-level mapping between images and the BIDS layout. This information and mapping are encoded into the ezBIDS_core.json, ezBIDS parses the fields in the JSON file and presents the information to the user in a series of steps (i.e. web pages). These steps allow ezBIDS, in partnership with the user, to bring the BIDS-mapping process to completion.

### Dataset Description

Once data have been uploaded and processed, the user interacts with the Dataset Description page. Alongside the ezBIDS_core.json, ezBIDS Core generates a BIDS dataset_description.json file, whose contents are displayed at this page. BIDS specifies that the fields in dataset_description.json must include basic information such as the dataset name and version of the BIDS standard used for the conversion. By default, ezBIDS Core pre-fills the BIDS version field. Additional fields are optional but recommended, such as authorship and acknowledgments, funding information for the study, license agreements specifying how the data may be used by others, and proper citation for using the dataset. ezBIDS offers users the ability to provide this information through the web page to better describe their dataset for the neuroimaging community (Figure S3).

### Subject and Sessions

Users then proceed to the Subjects and Sessions page, containing subject (and session, if applicable) entity labels. Modifications can be done manually or by choosing one of the options provided by ezBIDS through the “Reset Subject Mapping” button, consisting of three options: *PatientName*, *PatientID*, and *Numerical* (Figure S4). Selecting *PatientName* uses the PatientName metadata for the subject label. Selecting *PatientID* uses the PatientID metadata for the subject label, which may contain different information than PatientName, despite the similar attribute names. Selecting *Numerical* will create numeric values as subject labels, in chronological order. Numerical values less than 10 are zero-padded (i.e. 01, 02, etc).

### Series Mapping

Data are organized by their unique series IDs, with BIDS information (*data type*, *suffix*, entity labels) detected from the ezBIDS Core displayed (Figure S5). Users may modify this information, which is then applied to all images with the same unique series ID. For example, a dataset containing 10 subjects where each subject contains a T1w anatomical image means that these 10 images have the same series ID. If a user adds “*mprage*” to the acquisition (acq) entity label of this series ID, then all 10 images with that unique series ID have the acq-mprage entity label applied. This is meant to alleviate time spent on modifications by the user; rather than having to apply the label individually to potentially dozens of images, the user only modifies one image with the unique series ID. Users have the ability to make changes to individual images upon reaching the Dataset Review page (see point **6** below).

An important aspect to note is how the run label is applied. If a dataset contains functional BOLD data, the images may all have the same unique series ID because they contain the same identifying metadata (*SeriesDescription*, *ImageType*, *RepetitionTime*, and *EchoTime*). Users are not required to set the run label at the Series Mapping stage, as ezBIDS automatically determines the run label and applies it for users to see on the Dataset Review page. It should be noted that the run label can be applied to all data types and suffixes, not merely functional BOLD images.

### Events

**Fig. 4:**
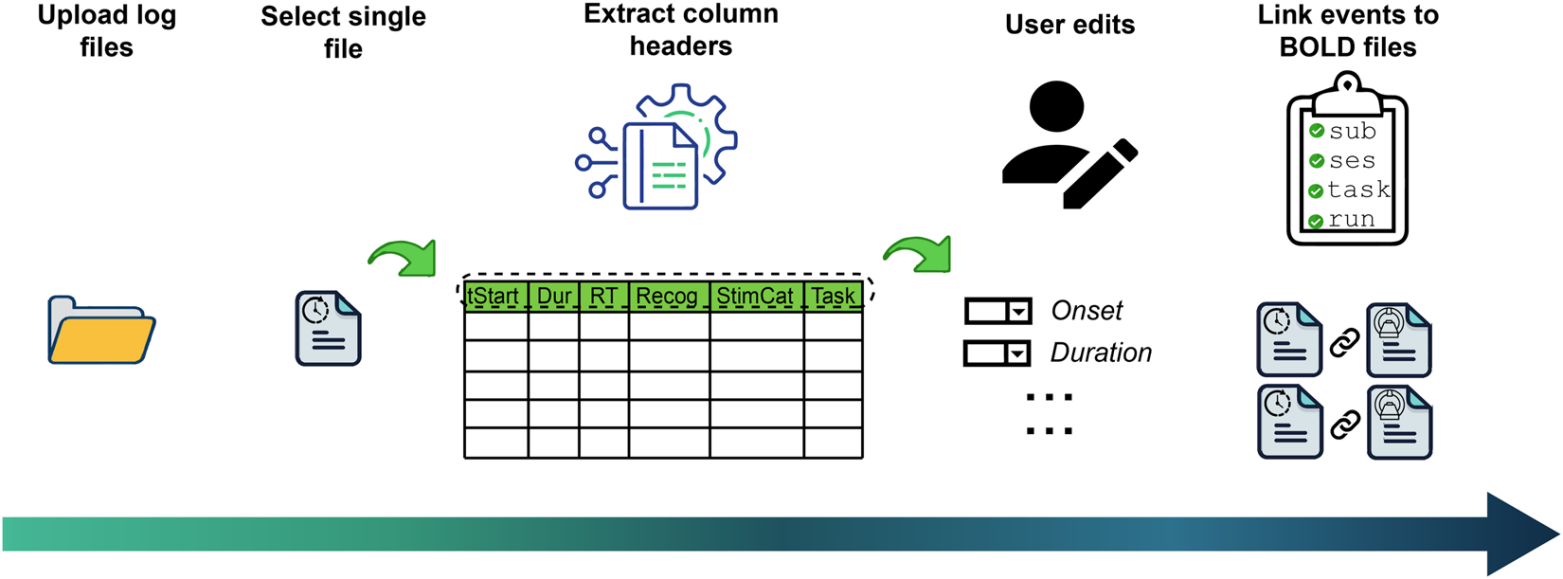
ezBIDS events conversion process. Once users have uploaded their task events timing files, ezBIDS performs a backend process to extract the column names based on the format of the uploaded files. The columns are then presented in a series of dropdown keys, enabling the user to specify which column name pertains to the BIDS task events columns. For time-based columns (e.g., onset), the user may specify “seconds” or “milliseconds” to note the unit of time for the recorded columns data. As BIDS requires time-based data to be in seconds, ezBIDS will convert data from millisecond to seconds, if so specified. Once the user has finished, ezBIDS applies the changes, converts the file format to TSV, and links the events files to the corresponding functional BOLD files by matching the sub, (ses, if applicable), task, and run entity labels. If ezBIDS cannot determine this link, unique random entity label values are provided (e.g., sub-001), which the user would then be able to edit. To enable greater accuracy in linkage, it is recommended that users specify these entity labels in the log file paths or as explicit column names.

To date, ezBIDS is the only BIDS converter that assists users in converting task events timing files from the experiment to the BIDS-specified *events.tsv* format that is necessary for analyzing task-based imaging data. The lack of this feature in other converters is likely due to the large variation in the formatting of timing files across researchers, labs, presentation softwares, and experimental paradigms. Although BIDS does not require task-based functional BOLD images to have corresponding *events.tsv* files, failure to do so results in task-based datasets that cannot be replicated. Given the unique structure of ezBIDS, which relies on a partnership between proposed conversions by machine learning algorithms and the revised conversions provided by the user, ezBIDS is able to alleviate conversion hurdles in task events timing files by providing an intuitive conversion mechanism that works well with a broad array of timing file formats (Fig. 4).

Upon reaching the Events page, users may upload task events timing files, assuming they have task-based (i.e., non-rest) functional BOLD images (Figure S6). Accepted timing file extensions are *.csv,.tsv*,.*txt,.out*, and *.xlsx* and do not need to be formatted in any specific manner. Certain experiment presentation tools export their task events timing files in a specific format, such as E-Prime^23^, which can be handled by ezBIDS. Once uploaded, ezBIDS extracts the column names, which are presented alongside the BIDS *events.tsv* columns. Users then select which columns of their timing files pertain to the *events.tsv* columns; this action is performed once and then applied to all timing files, as it is assumed that all timing files for a scanning session will have the same formatting (Figure S7). Additionally, time-based *events.tsv* columns (*onset*, *duration*, etc.) require values to be in seconds; however, some researchers output timing information in milliseconds. ezBIDS, therefore, provides the option for users to select whether or not their column values are in milliseconds and, ultimately, converts the values to seconds.

Once the crucial columns are identified, ezBIDS aims to link each timing file to its corresponding functional BOLD task image. This is done by looking for convergence between four BIDS entity labels: sub, (ses, if applicable), task, and run. ezBIDS assumes a one-to-one mapping of timing files to functional BOLD images, meaning that ezBIDS examines column headers or the timing file path name for information to identify appropriate entity labels for each timing file to map the timing file to the appropriate image (e.g., “Subject”, “Sub”, “Subj”). If a proper mapping to an image is determined, the timing file is given the same *SeriesNumber* as the corresponding BOLD data so that they will be displayed alongside each other on the ezBIDS web page. Mapping between timing and BOLD data can be rendered more efficient and require less user intervention by uploading timing files that contain explicit information about subject, (session, if necessary), task, and run either in their file paths (e.g., *sub-01/ses-01/task-bart_run-01*) or in their file columns.

Following at least one of these recommendations will result in the most efficient and automatic mapping possible. Should the mapping be imprecise – for example, a timing file cannot be automatically mapped to a specific subject – ezBIDS provides a placeholder value (e.g., sub-XX1), which the user then modifies to correct the mapping. Once modified, the web page is updated so that the *events.tsv* file is displayed alongside its corresponding functional BOLD image.

### Dataset Review

At this stage of the ezBIDS conversion process all files are displayed by their subject (and session) entity label(s), and ordered chronologically (Figure S8). Unlike the Series Mapping page, where a single user modification affects all files in the group, here users make adjustments to specific files if need be. For example, a user may be aware that a specific subject’s functional BOLD image is unusable due to excessive motion and do not wish to have the specific image converted to BIDS. The user has the ability to navigate to the data in question and exclude it from further BIDS conversion or add an entity label indicating the issue (e.g., acq-highMotion). For additional guidance, ezBIDS provides screenshots of each image and enhanced visualization examination through the NiiVue (https://github.com/niivue/niivue) plugin, a web-based neuroimaging data visualization tool that runs on most operating systems and web devices.

**Fig. 5:**
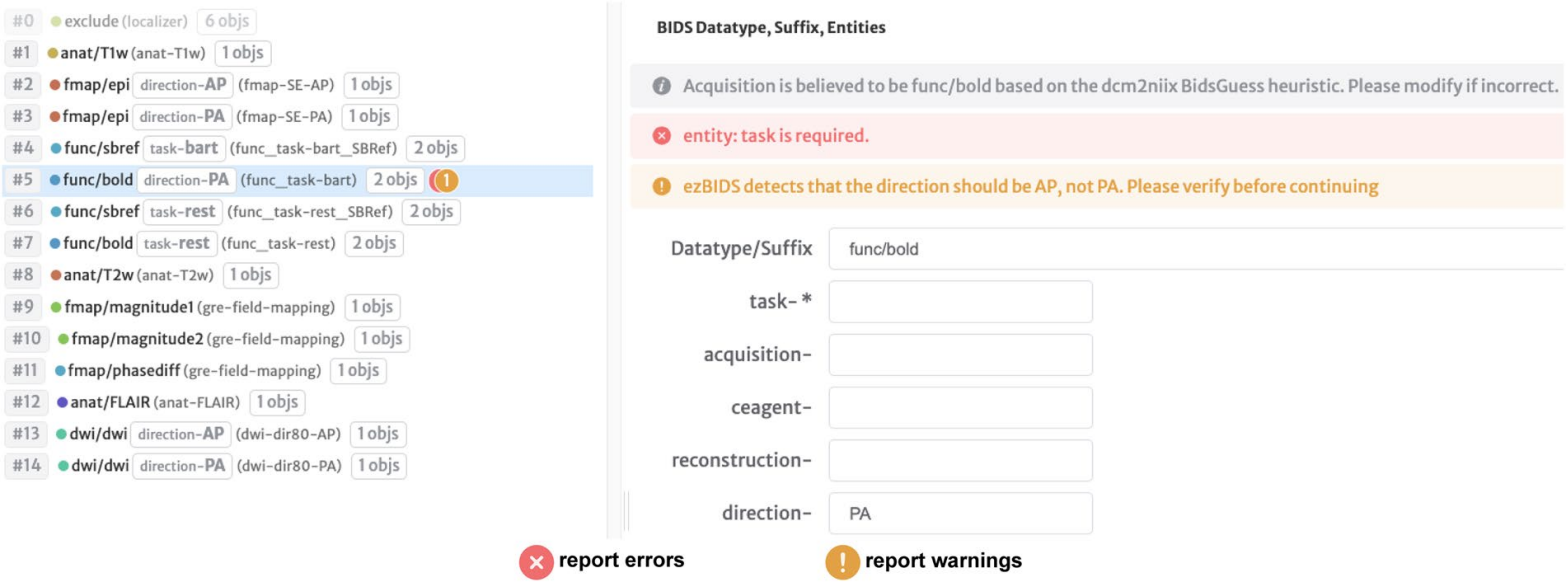
ezBIDS errors and warnings for compliance and improved quality of BIDS datasets. ezBIDS alerts users to BIDS validation errors and provides additional guidance to BIDS validation via custom warnings, to ensure BIDS compliance and to provide recommendations for improved curation of datasets. **Errors**. Marked by an “x” symbol (red). Errors identify details that prevent the dataset from being BIDS compliant, identical to BIDS validator errors. These are displayed in red and must be rectified by the user before proceeding. In the example, the task entity label is missing from the *func/bold* sequence, which is required by BIDS. ezBIDS therefore flags it as an error to alert the user that this must be addressed. **Warnings**. Marked by an “!” symbol (gold). Warnings offer recommended changes to the current BIDS structure that would improve the quality and curation of the dataset, and are displayed in dark yellow. Unlike errors, ezBIDS warnings are different from the BIDS validator warnings; ezBIDS warnings are unique recommendations presented to users as a way to improve the quality of details and metadata that the final BIDS datasets will be stored with. In the example, the user specifies the phase encoding direction (direction) entity label as “PA”, when in reality, ezBIDS knows that the correction label should be “AP”. Such warnings alert users to potential mistakes that might overwise pass BIDS validation. ezBIDS is agnostic with regard to how users respond to these warnings, meaning that users can ignore warnings and proceed if they so choose, since warnings do not preclude BIDS compliance.

On the *Dataset Review* and *Series Mapping* pages, ezBIDS performs quality assurance checks to ensure adherence to the BIDS standard by executing the *bids-validator* and alerting users if some aspect of their data warrants closer inspection, specifically, validation errors and warnings (Fig. 5).

Validation errors are distinct from validation warnings. Errors are presented in situations where modifications or lack of information do not align with the BIDS specification, requiring correction before being allowed to progress. For example, entity labels cannot be alphanumeric; if a user attempts to specify the acquisition (acq) entity label as *0.8 mm* then this is flagged by ezBIDS as an error. Users are unable to progress to the next page until all errors have been addressed. Warnings are generated in situations where the entity labels or data structure might not be proper; however, unlike validation errors, user intervention is not required for warnings and users may progress to the following page without addressing them. For example, 4D data sequences with a limited number of volumes (as determined by ezBIDS) are excluded (i.e., set to exclude) from conversion, as ezBIDS determines these sequences do not contain enough information to be of use to researchers. Users may elect to override this ezBIDS suggestion (i.e. un-exclude the sequence from conversion), however, ezBIDS will alert the user to the low number of volumes. This ensures that users are aware of their actions, but are not required to follow these recommendations. These warnings merely act as suggestions to improve the quality of the BIDS dataset.

### Pseudo-anonymization and defacing

Pseudo-anonymization is necessary for sharing data-sharing purposes^24^. ezBIDS provides a web-interface to run software that can remove voxels containing face information from anatomical images (Figure S9). Two defacing options are supported by ezBIDS: *Quickshear*^25^ and *pydeface*^26^. *Quickshear* requires a skull-stripped brain mask as an input, which ezBIDS generates with the *ROBEX* tool^27^. The recommended option is to use the *ROBEX* + *Quickshear* method on ezBIDS, as its performance is comparable to *pydeface*, but reaches completion in a noticeably shorter time. Before defacing begins, ezBIDS executes FSL’s *reorient2std* function^28^ on each anatomical image to ensure that the defacing tools do not perform suboptimally due to uncommon image orientations (e.g., if an image were to be uploaded oriented Anterior-Left-Superior (ALS), it would be reoriented to Right-Posterior-Inferior (RPI) in order to match the MNI152 standard template orientation). While this procedure changes the anatomical orientations, this poses no issues for users, particularly ones interested in processing and analyzing their BIDS data with processing tools like *fMRIPrep* that automatically reorient anatomical images to the RAS + orientation^29^. It should be noted that these defacing options are considered pseudo-anonymization steps due to advances in machine learning that could potentially result in re-identification of faces even from defaced anatomical images^24^. Regardless, this should not deter users from performing defacing to better conceal participants’ identity.

### Participants Information

For each non-excluded subject, ezBIDS attempts to report basic phenotype information (age, sex) from the images’ metadata (Figure S10). Users may populate these fields and/or provide additional participant information (e.g., handedness), which may be used as covariates in analysis models. Users may also choose not to include these fields and/or remove them.

### BIDS dataset validation and download

All information generated by ezBIDS and modified by users is assembled to provide the data in the finalized BIDS directory structure and file naming convention (Figure S11). Users can choose to share their unique and private ezBIDS URL with collaborators to ensure that the data standardization or quality is satisfactory (e.g., verifying that the entity labels used are agreed upon by the research team). Lastly, ezBIDS executes the *bids-validator* to ensure that the finalized dataset is BIDS-compliant, and displays the validator output to alert users to any lingering errors that may cause their data to be non-BIDS-compliant. Users are encouraged to address any displayed errors, but not prevented from saving the finalized dataset should the *bids-validator* find the dataset non-compliant. ezBIDS allows the output of non-compliant BIDS datasets, with the expectation that users will resolve the error(s) afterwards. If the user is satisfied with the final dataset, they have the option to download their BIDS-compliant dataset to their personal computer/server or upload the data to open repositories such as OpenNeuro.org^13^ or brainlife.io^14^. Lastly, users have the option to download an ezBIDS template file (ezBIDS_template.json) that can be uploaded with subsequent data to alleviate time spent on edits and modifications. This feature is discussed in the section below.

#### ezBIDS templates facilitate standardization of repeated usages

Once ezBIDS has guided the user to map the data into a BIDS-compliant dataset, they have the option to download an ezBIDS template file (ezBIDS_template.json) for future use. ezBIDS templates are a convenient way to reduce the work needed to upload scanning sessions subsequent to an initial one. The template contains all the information ezBIDS needs to map entire DICOM sessions (similar to the one just mapped) into corresponding BIDS sessions. The template also includes any customization or modifications made by the user when the template was created. For example, when users are collecting data for a study, they routinely collect data using the same (or very similar) scanning sequences in multiple subjects (dozens or hundreds of subjects). The template can be downloaded by clicking the blue “*Download ezBIDS Template*” button on the “*Get BIDS*” page.

ezBIDS templates allow reusing early scanning sessions as templates for later scanning sessions and reduce the amount of manual editing needed to pass the ezBIDS andBIDS validation. The ezBIDS_template.json file can be uploaded with a new scanning session, ezBIDS then detects the presence of this file and uses the information in the file to apply the edits and modifications made in the previous session to the current session. The information contained in the ezBIDS_template.json file is applied on the following pages: “Dataset Description”, “Subjects/Sessions”, “Series Mapping”, “Events”, and “Participants Info”. Uploading a ezBIDS_template.json file does not mean that ezBIDS will automatically proceed from start to finish, users will still need to review and approve the information automatically mapped. But the process is quick and ensures that all information is compliant.

#### Guided BIDS metadata capture

A key feature of the BIDS standard is the support provided to metadata management via sidecar files. BIDS metadata sidecars describe properties of the imaging data, such as detailed acquisition parameters, scan parameters or similar. ezBIDS, like most BIDS conversion tools, relies on dcm2niix^21^ to extract relevant metadata from the DICOM header. However, the BIDS standard is expanding to non-traditional MRI data and other imaging modalities (such PET, EEG and MEG). Because of the growth of the BIDS standard, metadata fields (required or recommended) are not all currently extracted by dcm2niix. For example, Arterial Spin Labeling (ASL) sequences require the BackgroundSuppression metadata field, and the corresponding BIDS specification supports that, yet such information is currently not extracted by dcm2niix. ezBIDS enables users to specify relevant metadata fields not handled by dcm2niix. These fields can be added using a web interface and are then injected into the BIDS metadata sidecar. Importantly, ezBIDS checks user inputs to ensure that the proper value type is specified. For example, the BackgroundSuppression field must contain a boolean (True, False) value; ezBIDS therefore alerts users if a non-boolean value is specified for this metadata field. Additionally, the BIDS specification necessitates certain metadata to be specified if another metadata field is specified. For example, with ASL sequences the BolusCutOffTechnique metadata becomes a required field if the BolusCutOffFlag metadata is provided. ezBIDS handles these dependencies and alerts users when these instances occur, to ensure thorough metadata compliance. While in theory this expands ezBIDS support to non-MRI modalities (MEG, EEG, NIRS, etc.), at the time of this article this feature has only been thoroughly tested and validated on MRI (including ASL) data, and soon to encompass PET and MEG sequences. This unique feature extends the ability of ezBIDS to handle any type of additional data modalities with or without dcm2niix support.

#### Web-security features of ezBIDS

As a web-based service that handles sensitive study participant imaging data, an important feature of ezBIDS is the secure implementation of its service stack. At the time of publication, ezBIDS runs on Jetstream2 Cloud, a HIPAA-aligned cloud computing infrastructure^30^. ezBIDS generates a unique key token for each new session that protects data from unwanted access. ezBIDS session token authentication is done through JSON Web Token (JWTs), which are encrypted using RS256 and revoked 3 days after signing. All ezBIDS sessions are anonymous, and stored data can only be accessed through an ezBIDS session URL with a unique key token. Data are stored for 5 days before being purged from the server. The communication between users and the ezBIDS web server is encrypted via https protocol using the *SHA256/TLS1.3* encryption algorithm (*TLS1.1* is excluded). Uploaded data is temporarily stored on a dedicated *ceph* Jetstream2 volume, and all backend services are executed on a private VM responsible for the receiving, handling, processing, and downloading of imaging data through the *nginx* proxy. As of Winter 2024, Jetstream2 provides a private subnet where the network traffic between the ezBIDS services can only be accessed by the member services. Users and collaborators with the proper URL have up to five days to work on their uploaded data, after which the data are purged from the storage system. Access to the private VM is restricted to only those who have the production ssh key as well as the administrators of the Jetstream2 Cloud who are trained IT professionals with proper HIPAA and system administration certifications. The activities within the VM are logged and monitored to detect and analyze improper use of the service.

#### Working with ezBIDS under Data Privacy Regulations

We note that ezBIDS uses cloud infrastructure with servers that at the time of this article are geographically located within the United States of America (USA). Although plans are being made to extend ezBIDS support using servers located in other regions, currently users outside the USA will be responsible to know their local regulations for data governance before using ezBIDS. For example, residents of the European Union may need to discuss with their local compliance officers whether they can be allowed to upload non pseudonymized DICOM or NIfTI files to ezBIDS. Below, we describe two approaches for ezBIDS users working with EU-resident imaging data.

The first option is to *pseudo-anonymize and deface* imaging data locally before uploading. Users may elect to anonymize their imaging data by using the *ba -y* option in the dcm2niix package. This removes potential identifying metadata information such as the AcquisitionDateTime, PatientID and PatientName. Then, users may deface the anatomical imaging sequences in their data, to remove or obscure facial features present in the data. These procedures enable greater adherence to GDPR guidelines.

The second option is to create an ezBIDS instance locally on a computer or server. This can be achieved by first forking or cloning the ezBIDS GitHub repository. The local computer or server must be Docker-enabled and have docker-compose installed. Once these conditions are met, users can simply execute the.dev.sh file (./dev.sh -d), located in the root level of the repository. This creates a local instance of ezBIDS that users may use, as files are accessed locally. Using ezBIDS in this manner prevents users from being able to push converted BIDS data to OpenNeuro.org or brainlife.io from the ezBIDs web-page; however, presents a localized way of accessing ezBIDS. For more specific instructions, see the Code availability section below. We note that this is an early feature of ezBIDS. An improved installation method for ezBIDS is planned.

#### Comparison of ezBIDS to other conversion tools

ezBIDS seeks to broaden the adoption of BIDS to a greater audience within the neuroimaging community; however, there exist other BIDS conversion tools with the same stated goal. Like ezBIDS, these conversion tools have their own unique workflows and implementations for transforming raw imaging data into BIDS-compliant datasets. Table 2 provides a comparison of ezBIDS to other commonly used conversion tools in the neuroimaging community along with a series of measures, though this is not an exhaustive list. Compared to other BIDS conversion tools, ezBIDS is the only tool with a web-interface, task events timing files conversion, QA checks, interoperability with OpenNeuro.org and brainlife.io, and a collaborative environment (via shareable ezBIDS session URL) with research team members that enables collegial BIDS conversion. ezBIDS is one of only two tools capable of embedding BIDS validation and being used without coding required from the user.

Additionally, ezBIDS accepts and handles a variety of BIDS datatype and suffix pairings, including quantitative MRI (qMRI) sequences^31^. These qMRI sequences are relatively new to the BIDS ecosystem, and as such, are not handled by most BIDS conversion tools.

## Discussion

Adoption of the BIDS standard promotes greater data sharing within the neuroimaging community by providing a common framework through which researchers can readily understand and utilize others’ data. Data sharing is of great importance to the neuroimaging community for addressing the longstanding issue of underpowered studies and improving reproducibility^3,32–34^. Despite the clear benefits of data sharing^35,36^ and a growing desire among researchers towards the implementation of FAIR principles^37,38^, such practices are not ubiquitous throughout the neuroimaging community. This remains problematic, given the enormous financial costs of conducting neuroimaging research and the plethora of imaging data that already exists. One likely reason for the disparity between the practice and the aspirations of researchers are the high expectations necessary to practice FAIR data principles: Findability, Accessibility, Interoperability, and Reusability^39^. Specifically, data adhering to the FAIR principles must be described with rich metadata and be stored in a openly available resource/repository (Findable), must be available to others (Accessible), be compatible with resources such as softwares, repositories, databases (Interoperable), and contain accurate and relevant metadata containing clear and sanctioned data usage policies (Reusable). Such adherence adds an additional burden on researchers, who must already contend with the demands of their own research programs, funding sources, and teaching responsibilities, among others. The current work lowers the barrier of entry to practice FAIR principles by providing the neuroimaging community with a user-friendly and intuitive platform for converting unformatted file structures of raw imaging data into the BIDS standard.

A standardized framework for brain imaging data provides an ecosystem of supported-software tools that ingest BIDS data to perform automated, standardized processing and/or analyses, known as BIDS-apps^34^. These tools aim to minimize the impact of variability in processing and analysis pipelines across labs and institutions, which can affect results and hinder reproducibility^3^. A recent study exemplified this issue by demonstrating that processing and analyzing imaging data using three common yet separate software packages can lead to statistically different findings^3,40^. Furthermore, BIDS-apps provide standardized pipelines for processing/analyses that require minimal human intervention, due to the expectation of BIDS-compliant data as input. Access to BIDS data ensures that researchers can leverage BIDS-apps to reduce variability and save time in their processing and/or analysis pipelines.

ezBIDS lowers barriers for researchers who desire to practice FAIR data principles by offering many features that are not available in other BIDS conversion tools. Importantly, the unique features offered by ezBIDS target users who are more interested in working with a user interface for their BIDS conversion, instead of using scripting or a command-line interface (CLI). Thus, a major strength of ezBIDS is that it does not rely on the CLI; however, it is possible that some researchers may consider this a limitation that hinders their ability to tailor ezBIDS to their specific needs. Future versions of ezBIDS may be developed further to include a CLI option for these users.

ezBIDS has been developed with MRI in mind, due to the authors’ extensive experience with this imaging modality. However, the expansion of BIDS over the past several years now includes additional imaging modalities, including PET and M/EEG, among others. Future versions of ezBIDS will seek to include conversion capabilities for these additional imaging modalities.

As a web-based service, thorough testing has been performed on the Chrome and Firefox browsers; however, the performance of ezBIDS has not been extensively vetted on others such as Safari, Microsoft Edge, Internet Explorer, and Opera. Future work will conduct testing on these additional web browsers.

As data-sharing practices continue to gain widespread acceptance, it is critical to create tools that lower the barrier of entry to the BIDS ecosystem (e.g., BIDS conversion tools and apps). This entails offering conversion tools that do not solely rely on command line interfaces or specific programming proficiency, thereby reducing the burden of learning a particular converter’s syntax and format. ezBIDS was designed with these considerations in mind, and as an innovative, open-source, web-based BIDS converter tool, aims to provide a streamlined and intuitive BIDS conversion experience to a broader range of neuroimaging researchers. As data sharing and adherence to the FAIR data principles become increasingly common, thanks in large part to BIDS, reproducibility issues in the neuroscience and psychological fields can begin to be addressed and will lay the foundation for new discoveries in the coming decades.

## Methods

The majority of the methods used by ezBIDS are described directly in the Results section above and in the supplementary materials.

ezBIDS began as a series of custom scripts designed to semi-automate the process from DICOM data to BIDS-compliant data. Over time, additional back- and frontend processes were incorporated to relay information to users on a webpage, enabling point-and-click modifications as opposed to requiring programming usage from users. To validate the ezBIDS workflow, collaborators from academic institutions across North America and Europe provided sample imaging data from a variety of scanner manufacturers (Siemens, Phillips, GE), consisting of various data types: anatomical (anat), functional (func), field maps (fmap), diffusion weighted imaging (dwi), perfusion (perf), and scanner parameters. This provided a robust set of validation data to ensure that ezBIDS could accommodate a broad range of MRI data. Shortly after, beta-testers began using the service with collected data to find bugs and suggest enhancements for improving the ezBIDS experience. Developers would meet with beta-testers to discuss their experiences using ezBIDS to ensure that ezBIDS provided them with an intuitive user interface for BIDS conversion. Development was performed in an environment separate from the beta-instance used by the beta-testers to ensure that ongoing work does not interfere with users’ interactions with ezBIDS.

ezBIDS has been validated using 30 shared datasets containing various types of data described in the BIDS specification from three scanner manufacturers. Errors and discrepancies in the mapping between DICOMs and BIDS structures for all these datasets and data types were used to inform updates on the ezBIDS code base and services.

### Supplementary information

Supplementary Information

## Data Availability

Imaging data was privately shared and cannot be publicly disseminated. However, a public validation dataset was collected at Indiana University and is available in a Figshare repository^41^. This data was approved by the Institutional Review Board (IRB) at Indiana University. The data contain identifiable images of the research participant (1st author), who provided informed consent for its dissemination.

## Code availability

All code is publicly available on our GitHub repository: https://github.com/brainlife/ezbids.
