# Supplementary material for: ezBIDS: Guided standardization of neuroimaging data interoperable with major data archives and platforms
Source: Sci Data. 2024 Feb 8;11:179. doi: 10.1038/s41597-024-02959-0 (PMC10853279; doi:10.1038/s41597-024-02959-0)
Supplement: Supplementary file 1 — Supplementary Information [file 41597_2024_2959_MOESM1_ESM.docx]

# **Table of Contents**

*Tables:*

Table S1 2

Table S2 4

Table S3 4

Table S4 6

*Figures:*

Figure S1 8

Figure S2 8

Figure S3 9

Figure S4 9

Figure S5 10

Figure S6 11

Figure S7 11

Figure S8 12

Figure S9 13

Figure S10 14

Figure S11 15

**Table S1**: Information pertaining to all functions used by the ezBIDS Core (ezBIDS_core.py).

| **Function** | **Input(s)** | **Output(s)** | **Description** |
| --- | --- | --- | --- |
| *find_cog_atlas_tasks* | *url*: string | *tasks*: list | Generates a list of all possible task names from the Cognitive Atlas API task webpage. |
| *correct_pe* | *pe_direction*: string  *ornt*: string | *proper_pe_direction*: string | Equivalent to fMRIPrep’s *get_world_pedir* (Esteban et al., 2019) function. Takes phase encoding direction and image orientation to correct *pe_direction* if needed. This correction occurs if *pe_direction* is in “xyz” rather than “ijk” format. |
| *determine_direction* | *proper_pe_direction*: string  *ornt*: string | *direction*: string | Takes [corrected] pe_direction and image orientation (from *correct_pe*) to determine the “dir-” BIDS entity label (e.g. AP, PA, RL, LR). |
| *modify_uploaded_dataset_list* | *uploaded_json_list*: list | *uploaded_files_list*: list  *exclude_data*: boolean  *config*: boolean  *config_file*: string | Filters the list of json files generated by *preprocess.sh* to ensure that the json files are derived from dcm2niix, and that they contain corresponding NIfTI (and bval/bvec) file(s). Additionally, Philips PAR/REC files are excluded, as they cannot easily be handled by ezBIDS. Furthermore, checks whether an ezBIDS configuration template file is provided. |
| *generate_dataset_list* | *uploaded_files_list*: list  *exclude_data*: boolean | *dataset_list*: list | Takes list of NIfTI, JSON, (and bval/bvec) files generated from dcm2niix to create a list of info directories for each uploaded acquisition, where each directory contains metadata and other dicom header information to help ezBIDS determine the identify of acquisitions, and to determine other BIDS-related information (e.g., entity labels). |
| *determine_sub_ses_IDs* | *dataset_list*: list  *bids_compliant*: boolean | *dataset_list*: list  subjects_information: list  participants_info: dictionary | Determines BIDS subject (“sub”) and session (“ses”) entity labels. |
| *determine_unique_series* | *dataset_list*: list | *dataset_list_unique_series*: list | Groups images into unique series IDs, based on having the exact values for 4 metadata fields: SeriesDescription, ImageType, RepetitionTime, and EchoTime. |
| *datatype_suffix_identification* | *dataset_list_unique_series*: list  *lookup_dic*: dictionary  *config*: boolean | *dataset_list_unique_series*: list | Assesses the key values from each image dictionary to determine the BIDS *data type* and *suffix* labels. |
| *entity_labels_identification* | *dataset_list_unique_series*: list  *lookup_dic*: dictionary | *dataset_list_unique_series*: list | Determines additional BIDS entity labels, depending on the *data type* and *suffix* pairing of the image, since specific pairs require certain entity labels. |
| *update_dataset_list* | *dataset_list*: list | *dataset_list*: list | Updates the *dataset_list* with BIDS labels. |
| *modify_objects_info* | *dataset_list*: list | *objects_list*: list | Adds ezBIDS specific labels (e.g. *section ID*), which are used for ordering purposes, and removes identifying metadata. |
| *extract_series_info* | *dataset_list_unique_series*: list | *ui_series_info_list*: list | Captures a subset of information that will be displayed on the ezBIDS webpage to help users understand their data. |
| *template_configuration* | *dataset_list_unique_series*: list  subjects_information: list  config_file: dictionary | *readme:* list  *dataset_description_dic:* dictionary  *participants_column_info:* list  *dataset_list_unique_series:* list  *subejcts_information:* list  *events:* dictionary | If an ezBIDS configuration template file is uploaded, parses contents for user modifications made on previous ezBIDS session and applies those changes to the current session, decreasing time spent on edits. |

**Table S2:** ezBIDS terms used for regex search pattern analysis of the SeriesDescription metadata for identifying BIDS information, primarily the *data type* and *suffix* labels.

| **Localizer keys** | *localizer* | *scout* |  |  |  |  |
| --- | --- | --- | --- | --- | --- | --- |
| **Fieldmap keys** | *fmap* | *fieldmap* | *spinecho* | *sefmri* | *semri* |  |
| **T1w keys** | *tfl3d* | *mprage* | *spgr* | *tflmgh* | *t1mpr* | *anatt1* |
| **T2w keys** | *t2* | *anatt2* |  |  |  |  |
| **FLAIR keys** | *t2spacedafl* |  |  |  |  |  |
| **Func keys** | *func* | *fmri* | *mri* | *task* | *rest* |  |
| **DWI keys** | *dti* | *dmri* |  |  |  |  |
| **DWI derivative keys** | *trace* | *adc* |  |  |  |  |

**Table S3**: Definitions of metadata fields (DICOM key attributes) and their usage in ezBIDS.

| **Attribute Name** | **Tag** | **Description** | **Usage in ezBIDS** |
| --- | --- | --- | --- |
| *Series Description* | (0008, 103E) | Description of the Series | Referenced in regex search patterns for terms (see **Table S1**) that can provide identifying BIDS information.  Used to group data into unique series. |
| *ImageType* | (0008, 2218) | Image identification characteristics | Used for identifying BIDS information if regex search patterns fail to produce findings.  Used to group data into unique series. |
| *Repetition Time* | (0018, 0080) | The period of time in msec between the beginning of a pulse sequence and the beginning of the succeeding (essentially identical) pulse sequence | Used in volume threshold calculation for functional BOLD data.  Used to group data into unique series. |
| *Echo Time* | (0018, 0081) | Time in ms between the middle of the excitation pulse and the peak of the echo produced (kx=0) | Used for identifying BIDS information if regex search patterns fail to produce findings.  Used to group data into unique series. |
| *Inversion Time* | (0018, 0082) | Time in msec after the middle of inverting RF pulse to the middle of an excitation pulse to detect the amount of longitudinal magnetization | Used to determine the inversion entity label for MP2RAGE and IRT1 anatomical data. |
| *Echo Number(s)* | (0018, 0086) | The echo number used in generating this image | Used to determine the echo entity label multi-echo data. |
| *Patient’s Name* | (0010, 0010) | Patient's full name | Used to help identify subject (and session) BIDS entities and organize data. |
| *Patient’s Birth Date* | (0010, 0030) | Birth date of the Patient | Used to help identify subject (and session) BIDS entities and organize data. |
| *Patient’s Sex* | (0010, 00400 | Sex of the named Patient | Information for BIDS’s participants.tsv file, which describes properties of the participants. |
| *Patient’s Age* | (0010, 1010) | Age of the Patient | Information for BIDS’s participants.tsv file, which describes properties of the participants. |
| *Patient ID* | (0010, 0020) | Primary identifier for the Patient | Used to help identify subject (and session) BIDS entities and organize data. |
| *Protocol Name* | (0018, 1030) | User-defined description of the conditions under which the Series was performed | Used for the same purpose at Series Description, but only if SeriesDescription isn’t present in DICOM. |
| *Receive Coil Name* | (0018, 1250) | Receive coil used. | Used to identify images’ BIDS entity label for field map RB1COR data |
| *Flip Angle* | (0018, 1314) | Steady-state angle in degrees to which the magnetic vector is flipped from the magnetic vector of the primary field. | Used to identify the flip BIDS entity label for anatomical VFA, MPM, MTS, and field map TB1EPI, and TB1DAM data. |
| *Acquisition Date* | (0008, 0022) | The date the acquisition of data that resulted in this image started | Used to help identify subject and particularly session BIDS entities and organize data if PatientID, PatientName and PatientBirthDate fields fail to produce identifying information. |
| *Acquisition DateTime* | (0008, 002A) | The date and time the data acquisition that resulted in this image started. | Used to help identify subject and particularly session BIDS entities and organize data if PatientID, PatientName and PatientBirthDate fields fail to produce identifying information. |
| *Series Number* | (0020, 0011) | A number that identifies this Series. | Helps organize data for each scan session chronologically, for display purposes on the ezBIDS web interface, and for QA checks. |
| *Study ID* | (0020, 0010) | User or equipment generated Study identifier. | Used to organize data by dataset. |
| *Delay Time* | (0018, 1067) | Delay time in milliseconds from trigger (e.g., X-Ray on pulse) to the first frame of a Multi-frame image. | Used to identify the “acquisition” BIDS entity label for field map TB1SRGE data. |

**Frequently Asked Questions (FAQ) about ezBIDS and data access control**

Dozens of users at multiple institutions have used ezBIDS for research and education contributing to the development of an effective system. Several of these early adopter users have asked questions that we believe can be of help to other users. Table S4 provides a summary of some of the most common questions with answers from the development team. In summary, ezBIDS is a set of microservices, software as a service (SaaS), a system that processes data without human intervention. The development team of ezBIDS can get involved with users’ data but only in rare situations when users request support. Yet, given the complexity and pace of the modern development life cycle, users should assume that the ezBIDS development team does not have the time to access the users data.

**Table S4: FAQ pertaining to data access and privacy when using ezBIDS.**

| **Question** | **Answer** |
| --- | --- |
| Where will my data be stored while ezBIDS is doing the BIDS conversion? | *As of Fall 2023 data to be converted to BIDS will be stored on a dedicated ceph Jetstream2 volume located in the United States and maintained by Indiana University and the University of Texas at Austin. ezBIDS makes use of Jetstream2’s private subnet such that data being processed on ezBIDS can only be accessed by the services required for BIDS conversion. All backend services are executed on a private Virtual Machines responsible for the receiving, handling, processing, and downloading of imaging data.* |
| For how long will my data be stored there before being deleted? | *Data are stored for no longer than 5 days and can be deleted sooner, upon user request. Data in this temporary storage are retained solely for the purposes of user support.* |
| While my data are being stored on the server, who will be able to access the data? | *The developers of ezBIDS will have access to the server that houses the data and would be able to access the data, upon user’s request and only prior to the end of the 5 days data storage policy.* |
| Will they be able to access the entire data set, or will they be restricted in some way? | *ezBIDS developers can have full access to the entire uploaded data, but do not access any of the data files unless a user requests support. Data access requests and permissions must be provided in writing to the project director and sufficient documentation may be requested to ascertain whether data access can be officially granted.* |
| Are users able to control data access on ezBIDS? | *No, the default policy is that no one accesses users’ data unless the project director has approved data access by direct communication with the users uploading the data. ezBIDS developers do not view the contents of data files unless needed and explicitly granted permission to do so. Users should expect that even if access to data is granted the developers in most cases will have not time to dedicate to resolving users’ issues by accessing the data.* |
| Are there any differences in how ezBIDS stores users data for example when downloading the BIDS data or pushing the data to OpenNeuro.org, brainlife.io? | *No, all data sets are treated the same, software services process the data without human intervention and regardless of user selections.* |
| If users’ data were collected under an IRB that states that the data will only be stored on university servers; will I be non-compliant if I use ezBIDS on these data? | The answer is “possibly, yes,” and it is such only if the IRB does not specify what it is meant by the phrase “to store data,” i.e., long term preservation. ezBIDS is not a long-term preservation system nor a data archive. ezBIDS stores users data, in most cases, outside of the users’ University servers for up to 5 days for the sole purpose to provide a service to the user and without human intervention or actions on the data. |
| Will my IRB consider this to be data sharing, given that the data are temporarily being stored on non-university servers? | Generally data sharing is between two humans or human groups. ezBIDS is a software service and no human receives, accesses or operates on the data, so technically the data is never shared. Yet, different IRBs make different decisions on this issue, and it will be important for the user to discuss this with their IRB prior to using ezBIDS, if the user believes concerns might arise from using the service. The ezBIDS team provides boilerplate text to assist the user in these discussions. |

| 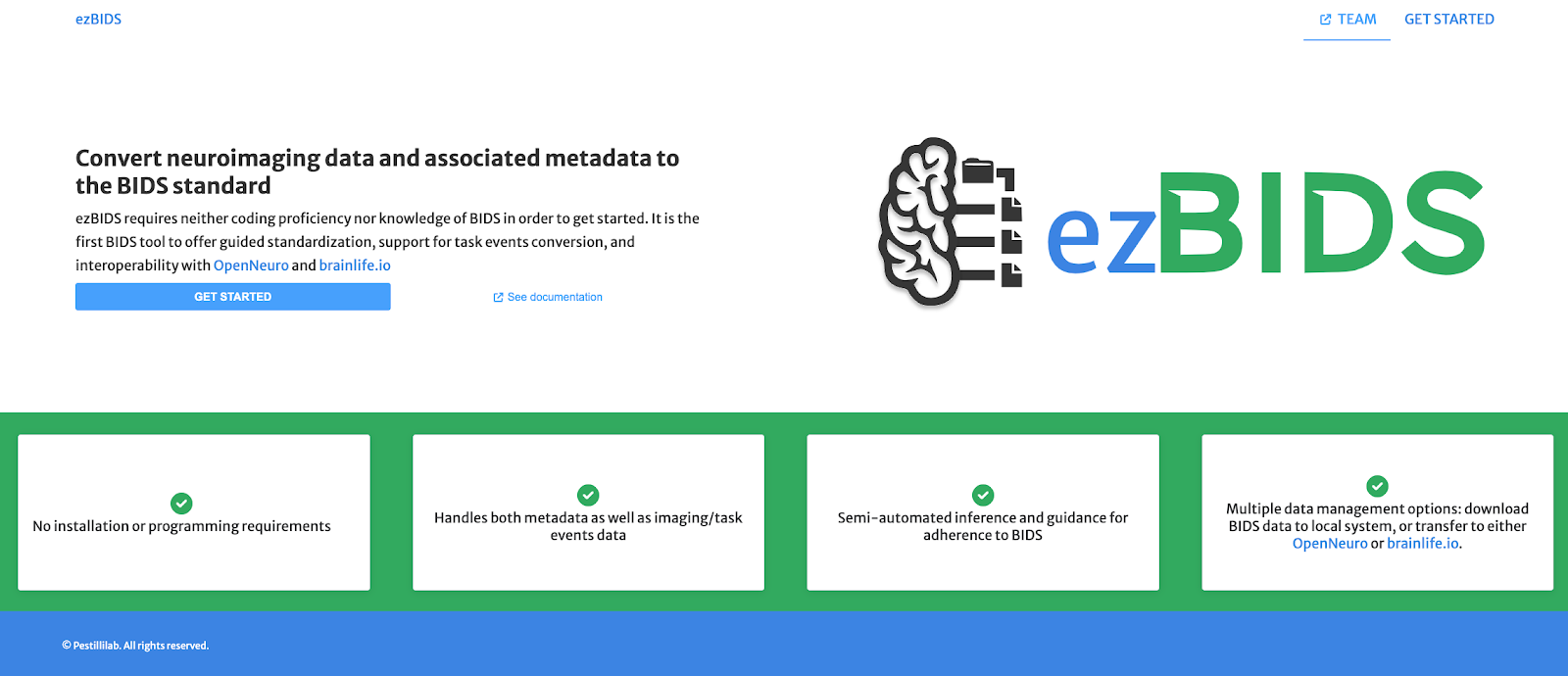 |
| --- |
| **Figure S1. ezBIDS homepage** |

| 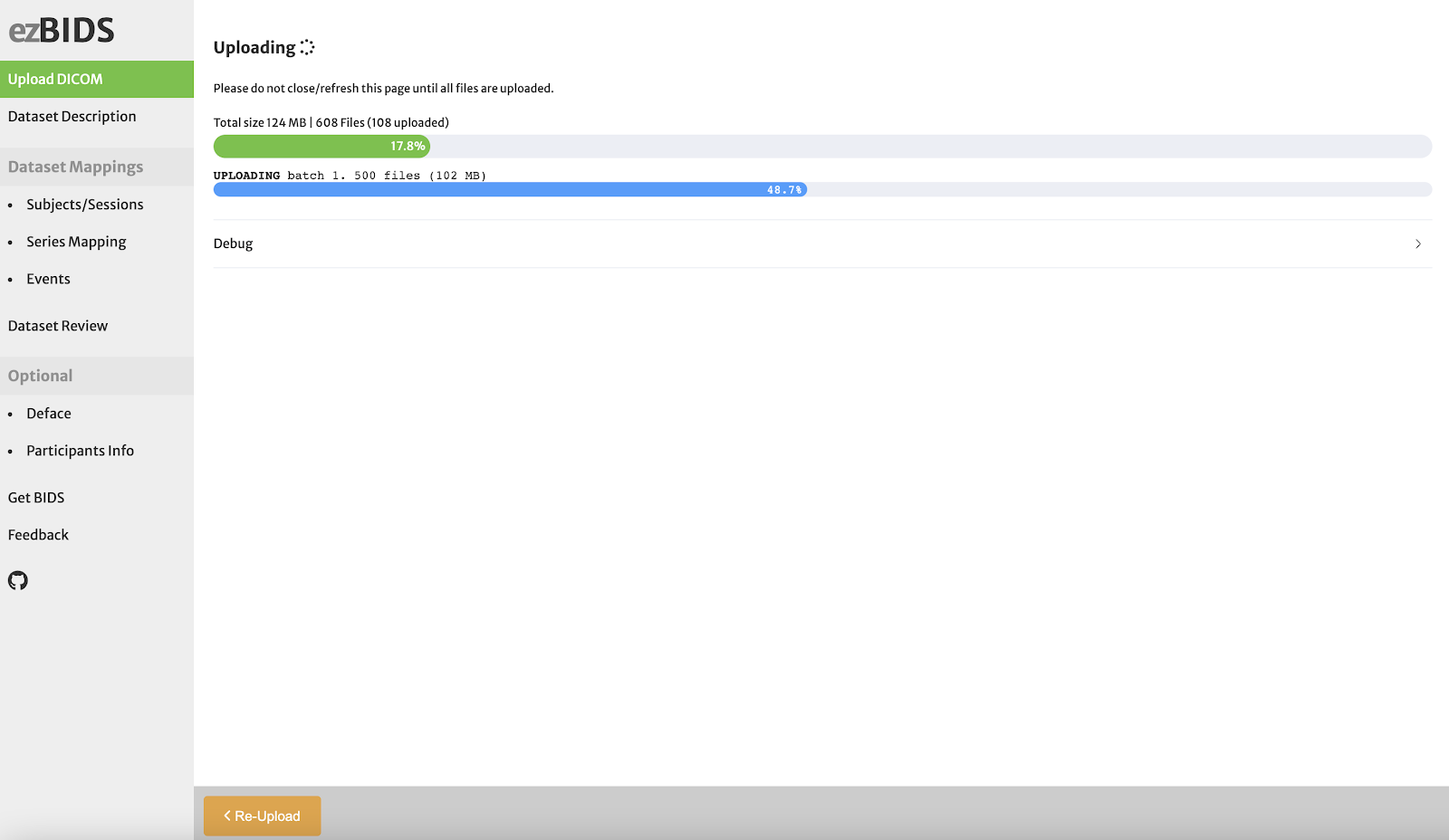 |
| --- |
| **Figure S2. ezBIDS upload web page.** |

| 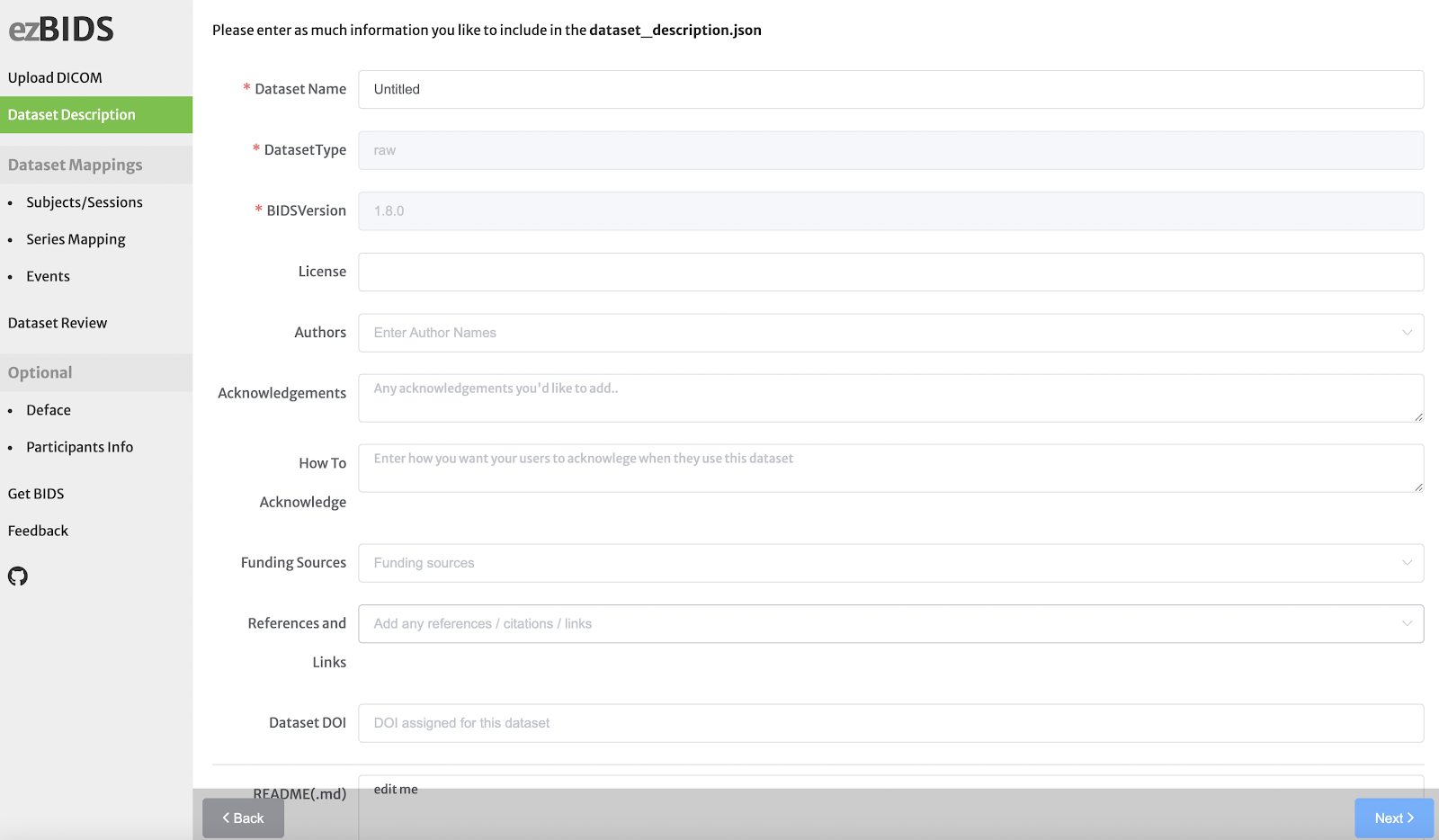 |
| --- |
| **Figure S3. Dataset Description web page.** |

| 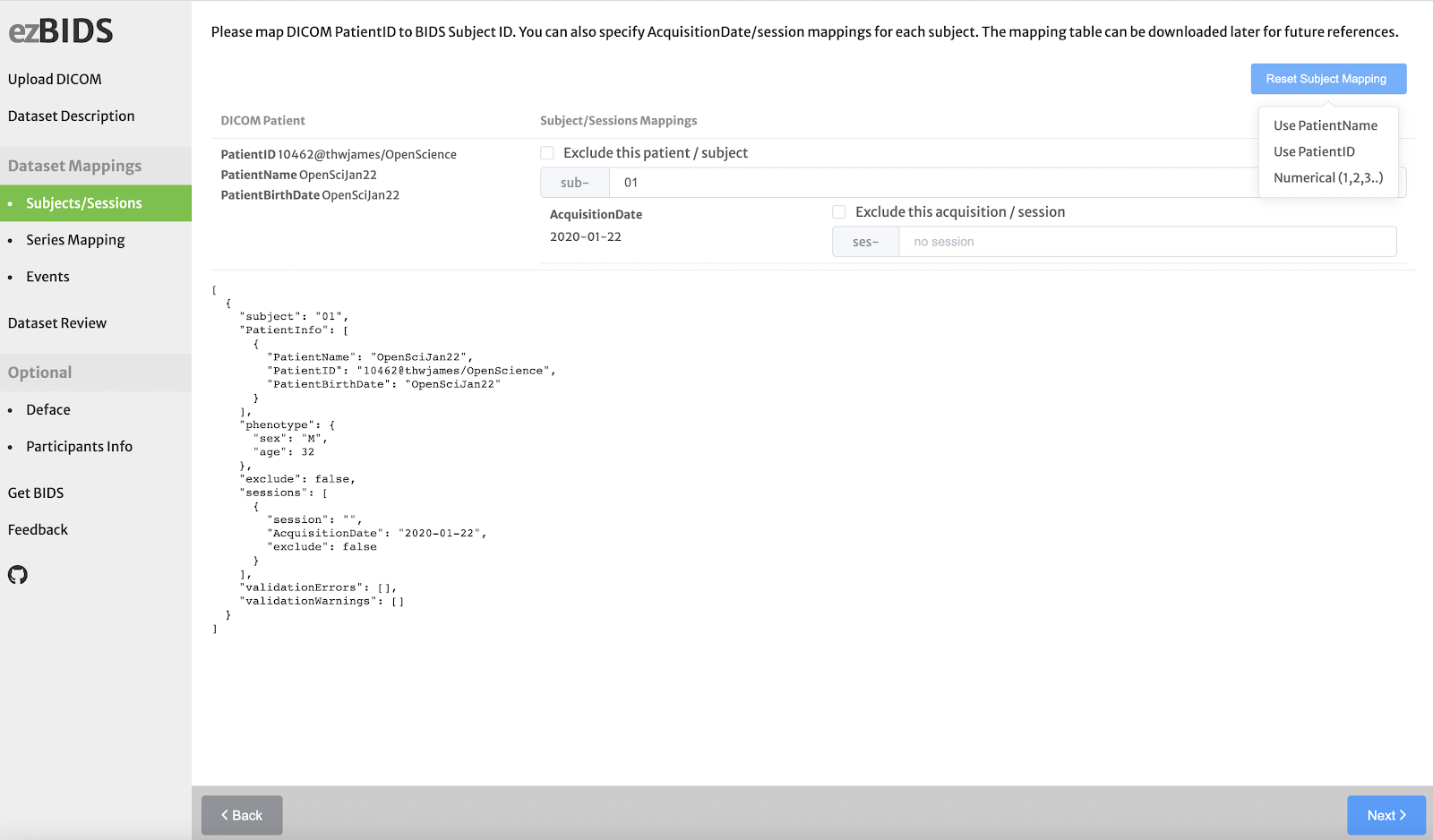 |
| --- |
| **Figure S4. Subjects/Sessions web page.** |

| 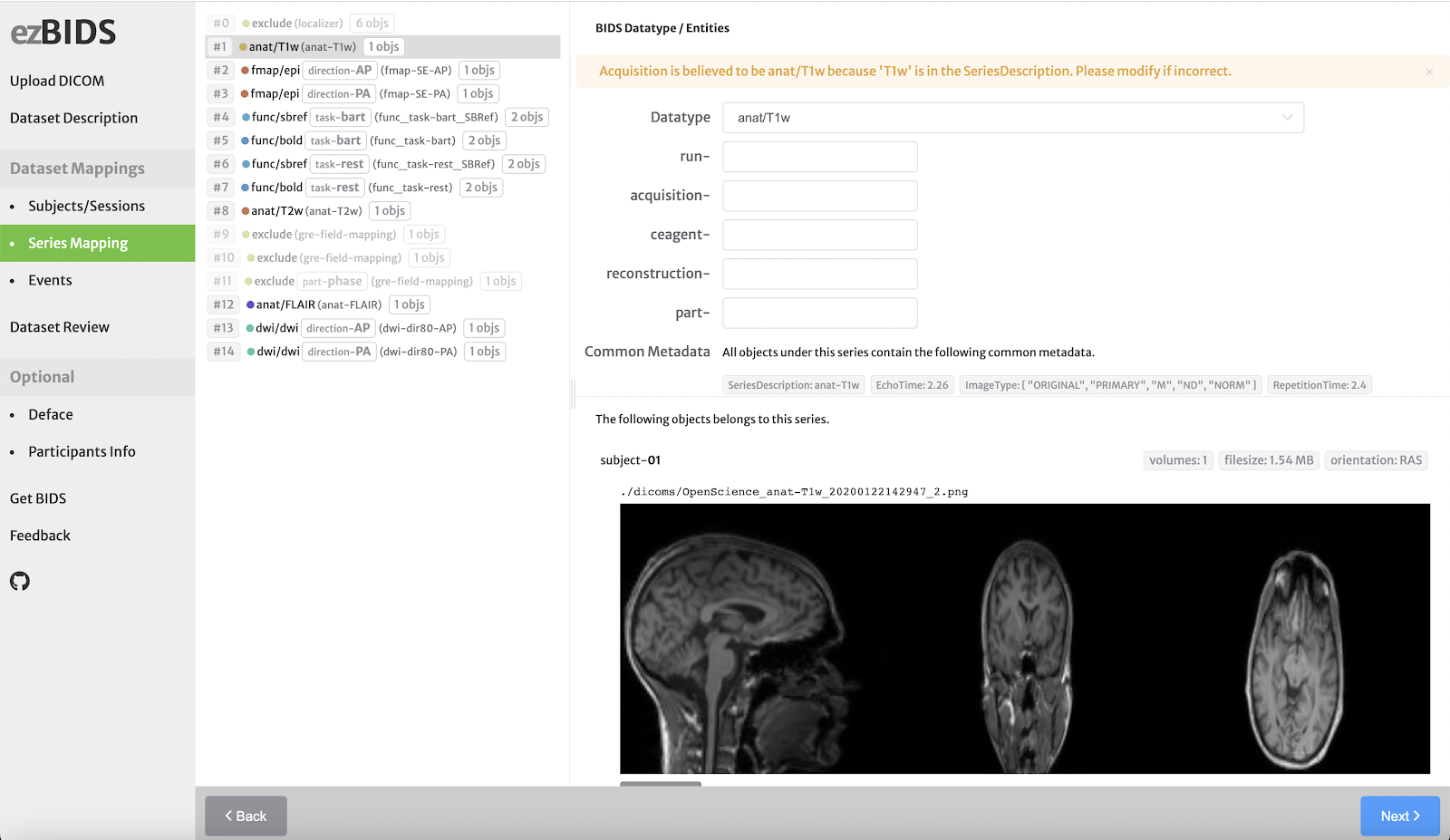 |
| --- |
| **Figure S5. Series Mapping web page.** |

| 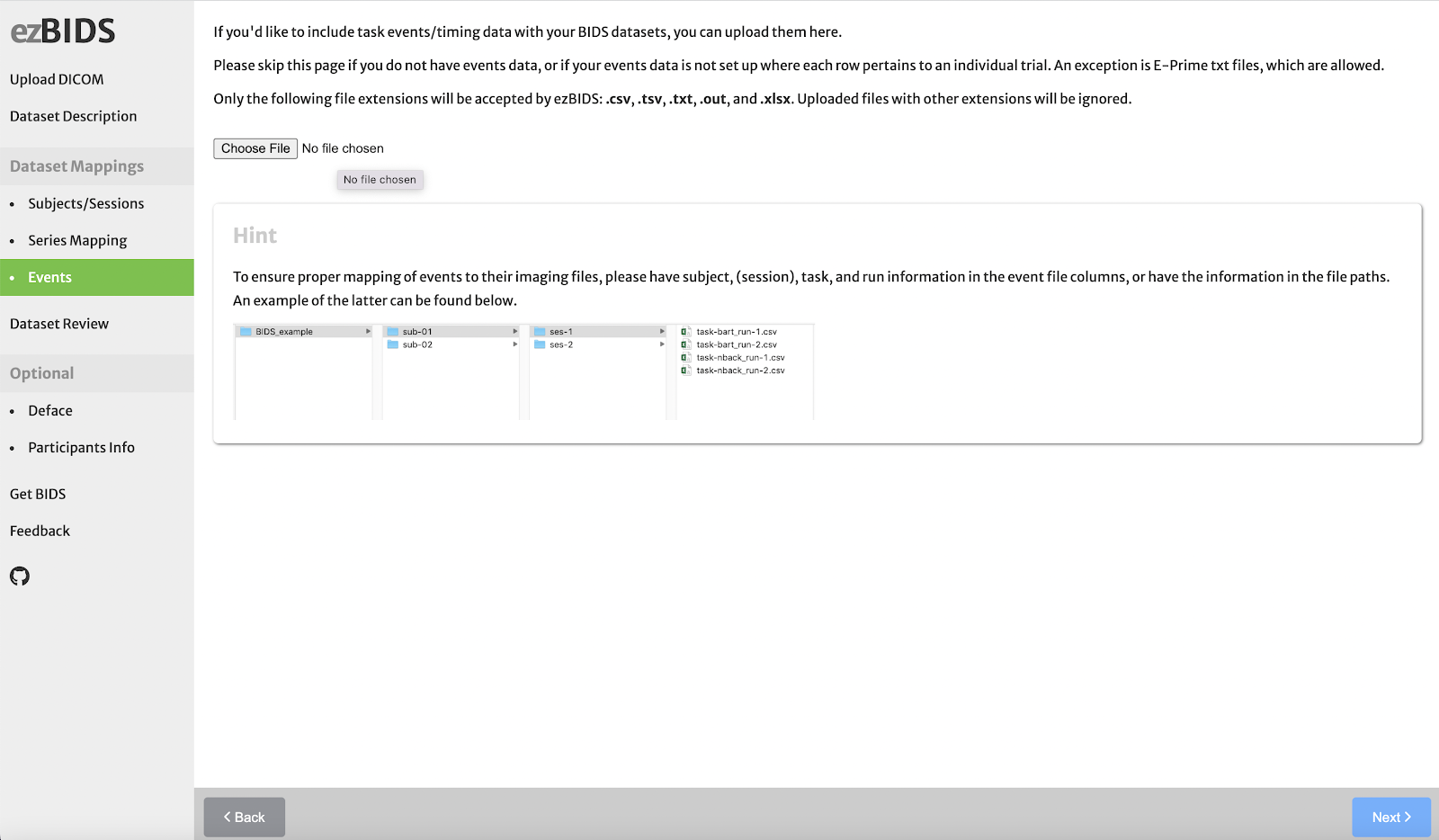 |
| --- |
| **Figure S6. Events web page.** |

| 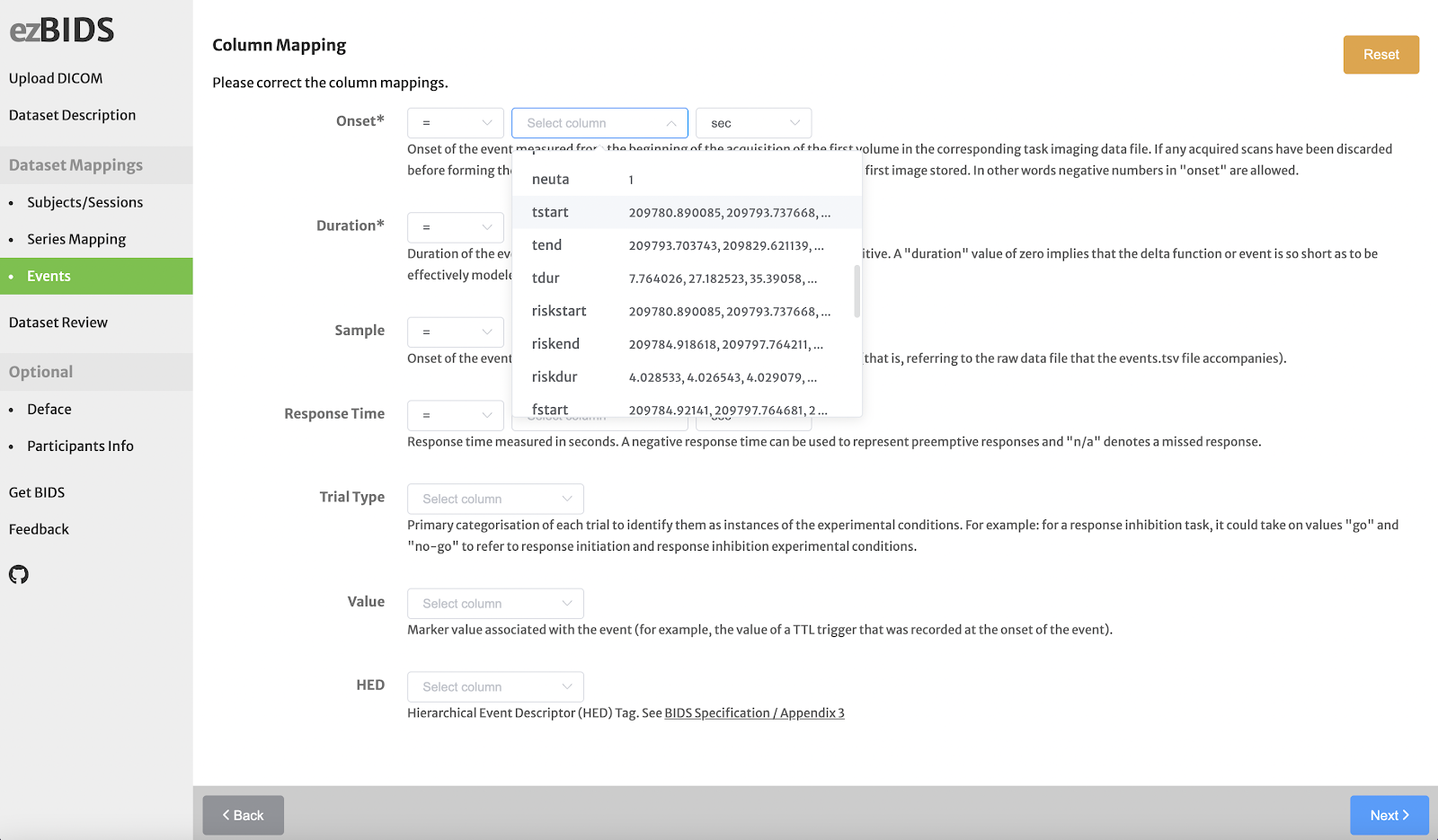 |
| --- |
| **Figure S7. Events Mapping web page.** |

| 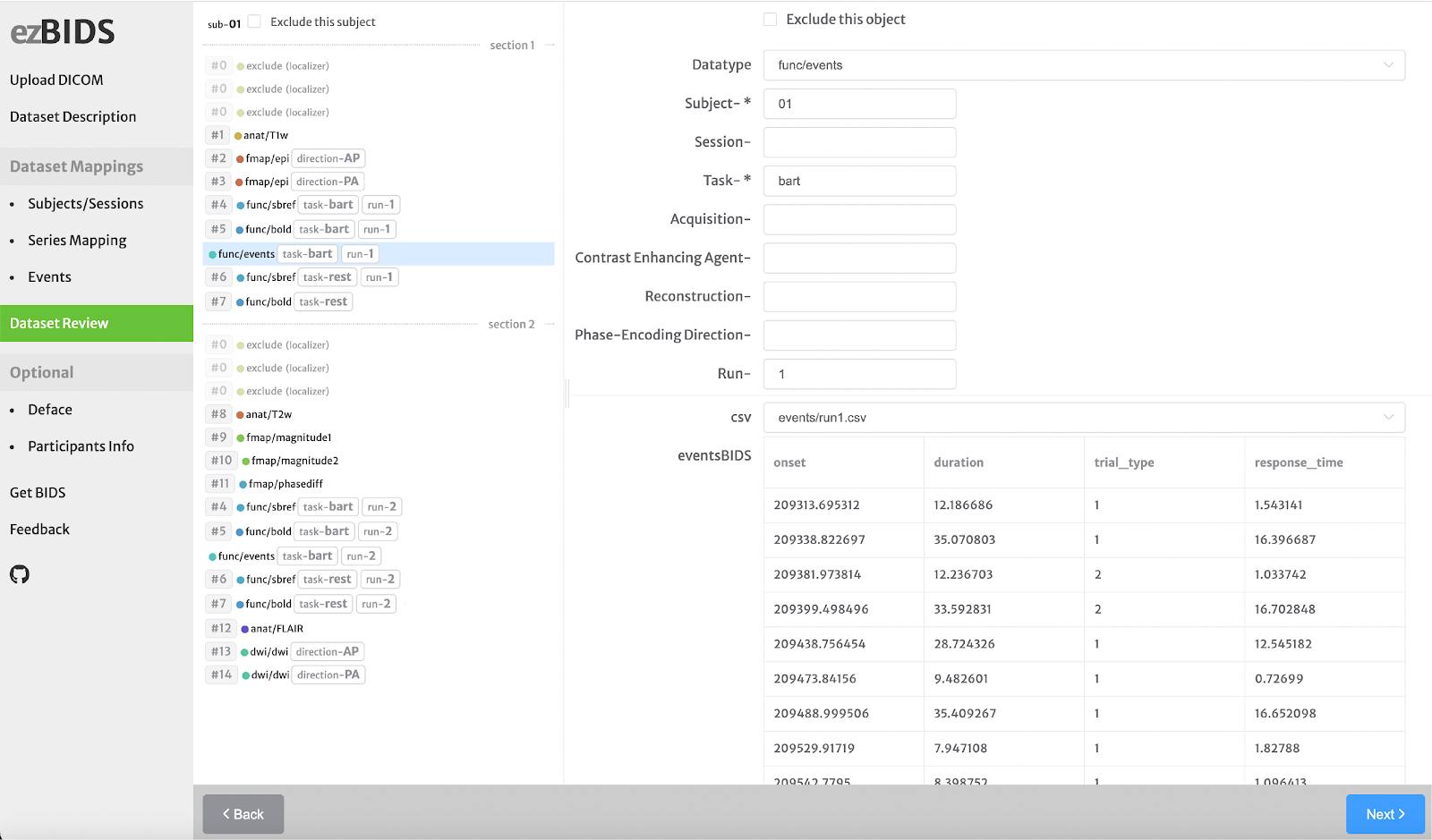 |
| --- |
| **Figure S8. Dataset Review web page.** |

| 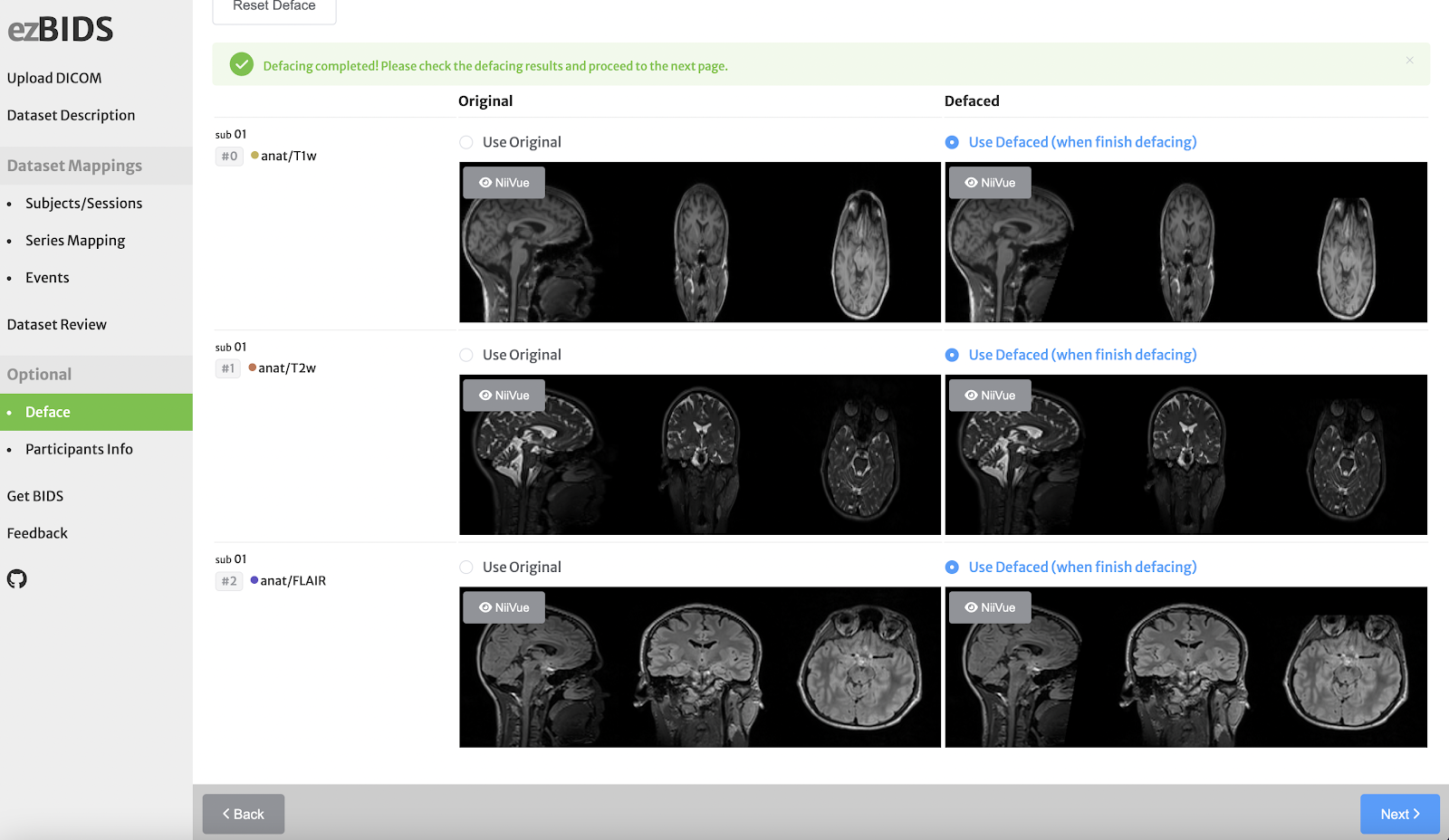 |
| --- |
| **Figure S9. Defacing web page.** |

| 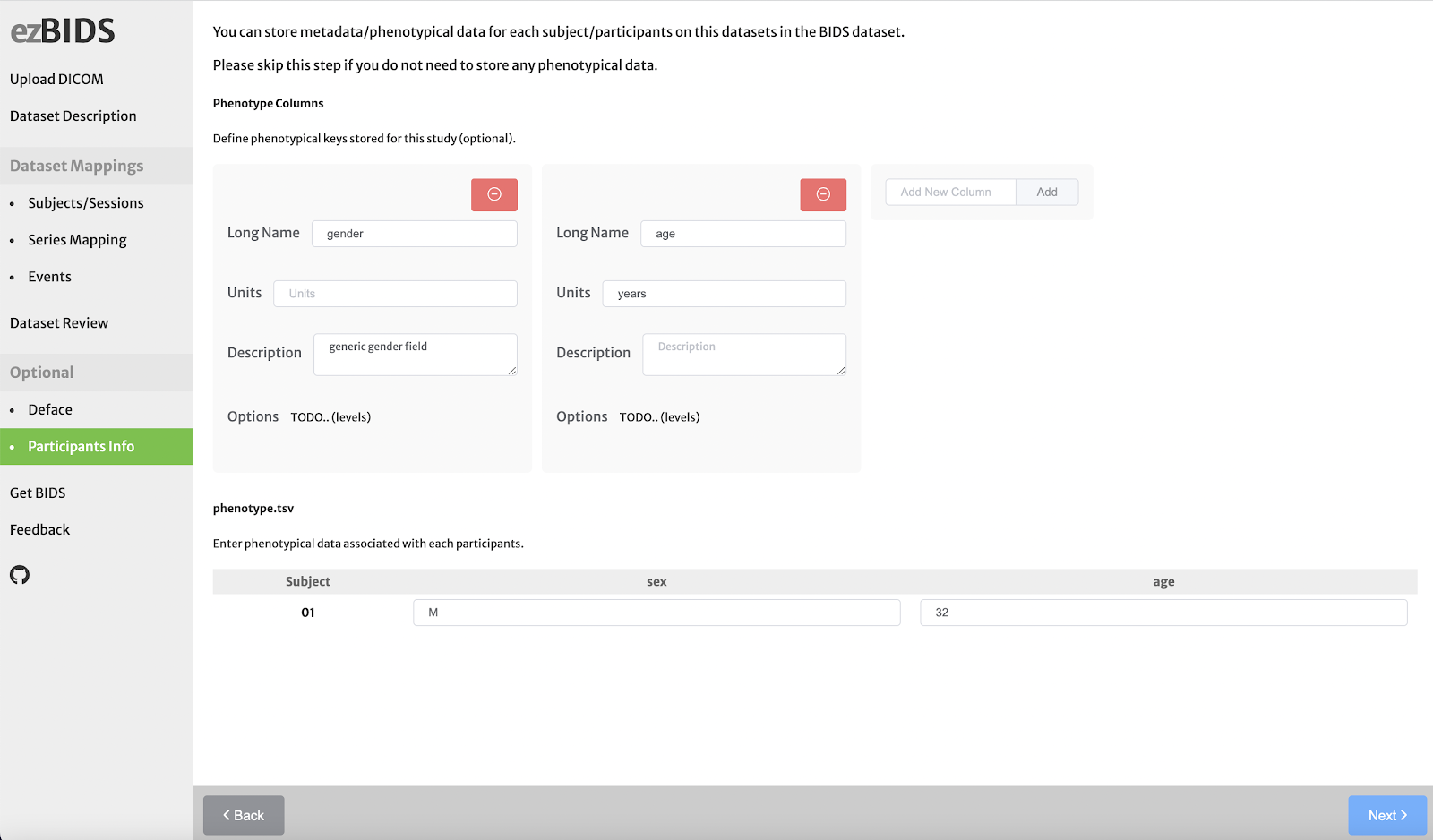 |
| --- |
| **Figure S10. Participants Info web page.** |

| 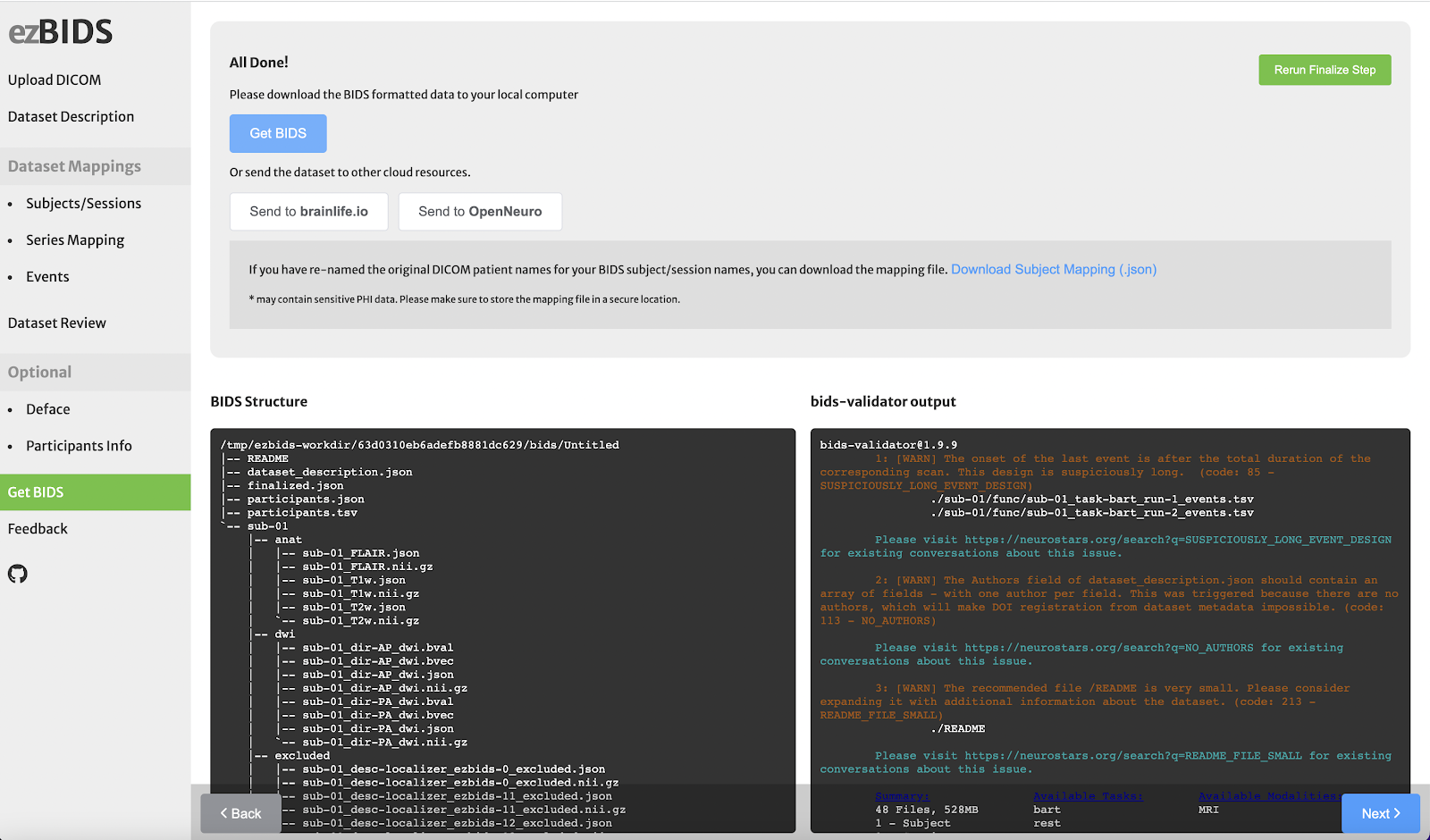 |
| --- |
| **Figure S11. Finalize web page.** |
